# Integrated transcriptomic and microbiomic analyses reveal mechanisms of Decapod iridescent virus 1 resistance in *Macrobrachium rosenbergii*

**DOI:** 10.3389/fimmu.2025.1611481

**Published:** 2025-05-26

**Authors:** Jingwen Hao, Yukun Jie, Zhibin Lu, Tiantian Ye, Jilun Meng, Cui Liu, Junjun Yan, Yutong Zheng, Zaijie Dong, Zhimin Gu

**Affiliations:** ^1^ Xianghu Laboratory, Hangzhou, China; ^2^ Wuxi Fisheries College, Nanjing Agricultural University, Wuxi, China

**Keywords:** Decapod iridescent virus 1, *Macrobrachium rosenbergii*, transcriptome, gut microbiome, disease resistance

## Abstract

Selective breeding for DIV1-resistant *Macrobrachium rosenbergii* is an effective strategy to mitigate aquaculture losses; however, the underlying resistance mechanisms remain poorly understood. In this study, approximately 2,300 prawns from 46 families were subjected to a DIV1 challenge test. Based on survival rate, viral load, histopathological observations, and viral gene detection in the transcriptome, one resistant family (R27-1) and one susceptible family (S2-2) were identified. Hepatopancreas transcriptomic (RNA-Seq) and gut microbiome analyses were conducted on samples at 0, 24, and 48 hours post-infection (hpi) from both families. A total of 144, 68, and 1,170 differentially expressed genes (DEGs) were identified at the respective timepoints. Three DEGs—including one corresponding to an uncharacterized lncRNA, an esterase E4-like protein, and a CUB-serine protease—were consistently differentially expressed at all timepoints. Transcriptomic data suggest that Melanogenesis, energy metabolism, and Steroid hormone biosynthesis pathways are associated with DIV1 resistance. Notable DEGs included *hemocyanin*, *cytochrome P450*, *alkaline phosphatase-like*, *Friend leukemia integration 1 transcription factor-like*, *cytochrome P450 9e2-like*, *interferon regulatory factor 4-like*, *dual* sp*ecificity protein phosphatase 10-like*, *trypsin II-P29-like*, and *cytochrome c oxidase subunit III*. In addition, the potential probiotic *Enterococcus casseliflavus* (relative abundance: 0.51% vs 0.03%) was more abundant in the resistant family, whereas *Lactococcus garvieae* (RA: 20.18% vs 70%) was enriched in the susceptible one. These findings highlight the combined contribution of host transcriptomic responses and gut microbial communities to DIV1 resistance. To the best of our knowledge, this is the first study to integrate transcriptomic and microbiomic analyses for investigating DIV1 resistance in *M. rosenbergii*. These findings provide novel insights into the host–pathogen interaction and offer valuable targets for selective breeding of DIV1-resistant *M. rosenbergii* in aquaculture.

## 1 Introduction

*Macrobrachium rosenbergii* (giant freshwater prawn) is a highly valuable aquaculture species, primarily cultivated in Asia, with China accounting for over 50% of global production in 2020 (1). In 2023, China’s production reached 196,374 tonnes, representing a 10.42% increase compared to the previous year (2). However, the rapid expansion of shrimp aquaculture has been accompanied by significant challenges, particularly disease outbreaks. Among these, Decapod Iridescent Virus 1 (DIV1) has become a major threat, causing high mortality rates and considerable economic losses in farmed populations (3). Thus, effective prevention and control strategies are urgently needed to sustain industry development. Selective breeding for disease-resistant broodstock offers a sustainable solution for disease control. With the advancement of molecular biology, genomic technologies now provide powerful tools to enhance and accelerate the breeding process (4). Identifying the key genes linked to disease resistance is the initial step in implementing marker-assisted selection (MAS) (5). Several genes related to disease resistance have been identified in aquatic animals. For example, a significant quantitative trait locus (QTL) associated with resistance to infectious pancreatic necrosis (IPN) in Atlantic salmon (*Salmo salar*) has been identified and is now being used in marker-assisted breeding to develop IPN-resistant fish (6, 7). In shrimp, polymorphisms in genes like *TRAF6* and *LvALF* have been associated with resistance to white spot syndrome virus (WSSV) (8–12).

Transcriptome sequencing combined with quantitative real-time PCR (qRT-PCR) in phenotypically distinct populations is an effective strategy for identifying genes linked to specific traits. For example, in *Litopenaeus vannamei*, transcriptomic comparisons revealed that genes like *myosin*, *myosin heavy chain*, and *chitinase*, involved in muscle growth and chitin metabolism, are upregulated in fast-growing families (13, 14). Similarly, in *Meretrix petechialis*, families with varying resistance to *Vibrio parahaemolyticus* showed differential expression of candidate genes such as *Big-Def*, *CTL9*, and *Bax* (15). Moreover, in *L. vannamei* affected by acute hepatopancreatic necrosis disease (AHPND), RNA-Seq identified 32 resistance-related DEGs, with 19 validated in progeny (16). Subsequent comparative analysis revealed 5,013 DEGs between *L. vannamei* resistant and susceptible families during *V. parahaemolyticus* infection, including 1,124 shared at 0 and 6 hpi, enriched in endocytosis, protein synthesis, and inflammation pathways, particularly mTORC1 signaling (17). In addition, differential expression of immune and metabolic genes such as *ChyA*, *SP*, *CRSTP*, and *PPAE2* between susceptible (P1) and tolerant (P2) *L. vannamei* populations further illuminated the molecular basis of AHPND tolerance and provided potential resistance markers (18). Consistently, in *Scophthalmus maximus*, resistant families showed more controlled inflammation and higher immune gene expression during early *Aeromonas salmonicida* infection, with many DEGs overlapping resistance QTLs (19). These findings collectively underscore the value of family-based transcriptomic comparisons in elucidating the molecular mechanisms underlying disease resistance in aquaculture species. However, despite these advances, the molecular basis of DIV1 resistance in *M. rosenbergii* remains largely unexplored, underscoring the need for in-depth investigation in this species.

In addition to host genetics, the intestinal microbiome has emerged as a critical factor in modulating disease resistance in aquatic animals. Commensal microorganisms on mucosal surfaces are crucial for defending the host against pathogen infections, and pathogen-induced changes in the intestinal microbiota have been widely documented across aquatic species (20–25). In crustaceans, the composition and function of gut microbial communities are tightly linked to immune responses and overall health. Dysbiosis following infection can influence disease outcomes. For example, WSSV infection in shrimp causes notable shifts in microbiota structure, and microbial indicators have been associated with disease severity (26, 27). Similar changes have been observed in *M. rosenbergii* following DIV1 infection (28). Changes in the gut microbiome were strongly linked to the severity of WSSV infection, and specific indicator taxa could serve as markers for assessing the health status of crustaceans (27). Studies also suggests that selectively bred *Flavobacterium psychrophilum*-resistant *Oncorhynchus mykiss* may harbor a more resilient gut microbiome compared to susceptible strains (25). The intestinal microbiome may regulate host immune homeostasis and inflammation, thereby improving resistance of *Cynoglossus semilaevis* to vibriosis through the microbe-intestine-immunity axis (23). These findings underscore the importance of the microbiome in disease resistance, highlighting the need to explore the relationship between the microbiome and DIV1 resistance in *M. rosenbergii*. By performing a comparative analysis of microbiome profiles across families with varying resistance levels, this study aims to better understand how microbial communities influence disease outcomes in shrimp aquaculture.

Although selective breeding offers a promising approach to improve DIV1 resistance, the underlying molecular and microbial mechanisms remain largely unknown. The hepatopancreas, a central organ for immune and metabolic regulation (29–31), is the primary target of DIV1 infection (32–34). In this study, we performed a comparative analysis of hepatopancreas transcriptomes and gut microbiome profiles in *M. rosenbergii* families with distinct DIV1 susceptibility, both before and after infection. By identifying DEGs and microbial taxa associated with DIV1 resistance, this work will deepen our understanding of the genetic and microbial factors underlying host tolerance, and provide a theoretical basis for marker-assisted selection in disease-resistant shrimp breeding.

## 2 Materials and methods

### 2.1 Pathogen

The DIV1 strain (GenBank accession number PQ724921) used in this study was isolated from naturally infected *L. vannamei* in Zhuhai, Guangdong Province, China. Tissue from DIV1-infected shrimp was homogenized in phosphate-buffered saline (PBS) at a 1:10 ratio using a high-throughput tissue homogenizer (SCIENTZ-48, NINGBO SCIENTZ BIOTECHNOLOGY CO., LTD, China). The resulting homogenate was subjected to two rounds of centrifugation: first at 3,000 rpm for 20 minutes at 4°C to remove large debris, followed by a second spin at 8,000 rpm for 25 minutes at 4°C to further clarify the sample. The supernatant was then passed through a 0.22 µm filter and stored at –80°C. qRT-PCR was performed to quantify DIV1 load, with primers listed in **Supplementary Table S1**.

### 2.2 Selection of susceptible and resistant families against DIV1

The *M. rosenbergii* families were obtained and maintained separately in Zhejiang Lanke Breeding Biotechnology CO., LTD. Before the formal experiment, five prawns were randomly selected from each family for pathogen screening, including tests for DIV1, infectious precocity virus (IPV), WSSV, infectious hypodermal and hematopoietic necrosis virus (IHHNV), *Vibrio parahaemolyticus* (Vp_AHPND_), and *Enterocytozoon hepatopenaei* (EHP), with the primers listed in **Supplementary Table S1**. The results indicated that all pathogen tests were negative. A challenge test was conducted to evaluate the susceptibility to DIV1 among *M. rosenbergii* families. A total of 46 families were selected for the assessment, with individuals averaging 1.82 g in body weight. After a 7-day acclimatization period, approximately 50 healthy prawns from each family were randomly selected, and each prawn was injected with 50 μL of DIV1 solution (virus concentration: 1.95 × 10^7^ copies/mL). The selection of the viral concentration was based on the results of a preliminary experiment, with the estimated median lethal dose (LD_50_) at 72 hpi and 96 hpi being 2.6 × 10^6^ copies/g of muscle tissue and 2.44 × 10^5^ copies/g of muscle tissue, respectively. Based on these findings, a virus concentration of 1.95 × 10^7^ copies/mL (5.36 × 10^5^ copies/g of muscle tissue) was selected for the formal challenge experiment, as it was capable of inducing moderate mortality and effectively distinguishing the resistance levels among different families. Throughout the experiment, water temperature was maintained at approximately 25 °C with continuous aeration. One-third of the water was replaced daily, and commercial feed was provided twice daily (morning and evening). Shrimp from each family were monitored every two hours and any dead individuals or uneaten feed were promptly removed. The exact time of death for each prawn was recorded, and cumulative mortality data were collected over a 14-day period. Based on survival rates and family numbers, one susceptible family (S2–2) and one resistant family (R27–1) were selected for further analysis.

### 2.3 Experimental design and sample collection

To minimize the influence of external environmental variables on subsequent observations, approximately 200 individuals from each of the two selected families were randomly selected, fluorescently labeled, and co-cultured in a single outdoor pond. After 73 days, 30 individuals from each family (approximately 15 cm in length and 50 g in weight) were randomly selected and acclimated in the laboratory for 7 days. To investigate the molecular immune mechanisms underlying the differences between DIV1-resistant and -susceptible families, a viral challenge experiment was conducted. As mentioned in section 2.2, the LD_50_-72 hpi was estimated to be 2.6 × 10^6^ copies/g of muscle tissue. Based on this finding, and taking into account the body weight of prawns used in this study, a corresponding infection dose was selected for the formal challenge. Accordingly, each prawn was intramuscularly injected at the third abdominal segment with 100 µL of DIV1 solution containing 9.3 × 10^8^ copies/mL, a concentration lower than the LD_50_ at 72 hpi. During the experiment, the water temperature was maintained at 25 °C with continuous aeration. Prawns were fed twice daily, and uneaten feed was removed promptly.

Tissue samples were collected at 0 h, 24 hpi, and 48 hpi. The selection of these time points was based on preliminary observations and insights from relevant literature. Specifically, 0 h was chosen to explore the global transcriptome and gut microbiota composition differences between the susceptible family S2–2 and the resistant family R27–1 under baseline conditions (19, 23). Based on the challenge experiment described in section 2.2, we observed that the highest population-level mortality occurred between 72 and 96 hpi. Since our primary focus was on host responses to DIV1 infection in the early stages, 24 hpi and 48 hpi were selected as key time points for comparative analyses. At each time point, hepatopancreas tissues were collected for transcriptome analysis (with 3 biological replicates per group). Intestinal tissues and contents were collected for microbial diversity profiling (n = 5 per group), and muscle tissues were collected for viral load quantification (n = 4 per group). Additionally, hepatopancreas tissues were collected at 48 hpi for histopathological examination using hematoxylin and eosin (H&E) staining, with PBS-injected shrimp serving as the negative control. The 48 hpi time point was selected for pathological analysis due to significantly increased viral replication (as indicated by viral load quantification), providing a critical window for assessing tissue damage and immune-pathological changes between the two families. For histological analysis, hepatopancreas tissues were fixed in 4% paraformaldehyde (Biosharp, China) and stored at 4°C. All remaining samples were rapidly frozen in liquid nitrogen and stored at –80 °C for subsequent analyses.

### 2.4 Histopathological observations

Histopathological section preparation and examination were conducted in accordance with standard pathological procedures. Briefly, hepatopancreas tissues fixed in 4% paraformaldehyde were sequentially dehydrated in graded ethanol solutions (70%, 80%, 95%, and 100%), followed by clearing in xylene. The cleared tissues were infiltrated with paraffin at 56–58 °C for 12 hours, transferred to fresh paraffin for an additional 12 hours, and then embedded. Paraffin-embedded tissues were sectioned into 3–5 μm slices using a microtome. Sections were mounted onto glass slides and dried at 60 °C for 30 minutes. After deparaffinization and rehydration, the sections were stained with hematoxylin and eosin (H&E), followed by dehydration, clearing, and mounting. The stained sections were observed under an optical microscope (Nikon Eclipse Ci-L, Japan) at different magnifications, and representative images were captured at 200× and 400×.

### 2.5 DIV1 load detection

To accurately quantify DIV1viral loads, a standard curve was established following the method described by Qiu et al. (35). DNA from the DIV1-ZH strain was extracted using the Genomic DNA/RNA extraction kit for aquatic animal pathogens (FAST) (DHelix, Guangzhou, China), and the concentration was adjusted to approximately 60 ng/µL. Quantitative analysis was performed using the primers and TaqMan probe listed in **Supplementary Table S1** with the Applied Biosystems™ QuantStudio™ 3 real-time PCR system (Thermo Fisher Scientific, USA). The reaction mixture consisted of 10 µL AceQ^®^ Universal U^+^ Probe Master Mix V2 (Vazyme Biotech Co., Ltd, Nanjing, China), 0.2 µL TaqMan Probe (10 µM), 0.4 µL DIV1-qF (10 µM), 0.4 µL DIV1-qR (10 µM), 8 µL ddH_2_O, and 1 µL template DNA. The thermal cycling conditions included denaturation at 37°C for 2 min, followed by 95°C for 5 min, and 40 cycles of 95°C for 10 s and 60°C for 30 s. DIV1 load was determined based on the standard curve, DNA concentration, and the obtained cycle threshold (Ct) values. Results were presented as the mean ± standard deviation (SD). Statistical differences in DIV1 loads between the susceptible and resistant families were analyzed using Student’s t-test, with significance levels indicated as *p* < 0.05 (*), *p* < 0.01 (**), and *p* < 0.001 (***).

### 2.6 RNA extraction, library construction, transcriptome sequencing, and read mapping

Total RNA was extracted from the hepatopancreas tissue using TRIzol^®^ Reagent (Qiagen, Germany) according to the manufacturer’s instructions. RNA quality was assessed using the 5300 Bioanalyzer (Agilent, USA) and quantified with the NanoDrop 2000 (Thermo Fisher Scientific, CA, USA). RNA was isolated, purified, and converted to cDNA according to the protocols provided by the respective reagent kits. RNA-seq libraries were prepared using 1 μg of total RNA per sample with the Illumina^®^ Stranded mRNA Prep, Ligation Kit (San Diego, CA). Briefly, messenger RNA (mRNA) was enriched using the polyA selection method with oligo (dT) beads and fragmented with fragmentation buffer. First-strand cDNA synthesis was followed by end repair, phosphorylation, and adapter ligation, as per the library preparation protocol. cDNA fragments ranging from 300 to 400 bp were size-selected using magnetic beads and amplified by PCR for 15 cycles. After quantification with Qubit 4.0 (Thermo Fisher Scientific, USA), the libraries were sequenced on the NovaSeq X Plus platform (PE150) using the NovaSeq Reagent Kit (Illumina, USA). Raw paired-end reads were trimmed and quality checked using fastp (36). Clean reads were subsequently mapped to the *M. rosenbergii* reference genome (GCF_040412425.1, https://www.ncbi.nlm.nih.gov/datasets/genome/GCF_040412425.1/) with HISAT2 (37) in orientation mode. The mapped reads were assembled using StringTie (38).

### 2.7 Differential expression analysis and functional enrichment

DEGs between the susceptible (S2-2) and resistant (R27-1) families were identified by quantifying transcript expression in transcripts per million reads (TPM), with gene abundance estimated by RSEM (39). Differential expression analysis was performed using DESeq2 (40), and genes with |log2FC| ≥ 1 and false discovery rate (FDR) < 0.05 were considered significantly differentially expressed. To further explore the biological significance of these DEGs, KEGG pathway enrichment analysis was performed, with significantly enriched pathways identified based on Bonferroni-corrected *p*-values < 0.05.

### 2.8 Sample DNA extraction, PCR amplification, and sequencing library construction

Total microbial genomic DNA was extracted from samples using the E.Z.N.A.^®^ Soil DNA Kit (Omega Bio-tek, Norcross, GA, USA) following the manufacturer’s instructions. The quality of the extracted DNA was assessed by 2% agarose gel electrophoresis, and its concentration and purity were measured using a NanoDrop2000 (Thermo Scientific, USA). The V3–V4 hypervariable regions of the 16S rRNA gene were amplified using the barcoded primers 338F (5’-ACTCCTACGGGAGGCAGCAG-3’) and 806R (5’-GGACTACHVGGGTWTCTAAT-3’) (41). Each 20 μL PCR reaction contained 10 μL of 2× Pro Taq buffer, 0.8 μL of each primer (5 μM), 10 ng of template DNA, and nuclease-free water to volume. The thermal cycling conditions were as follows: initial denaturation at 95°C for 3 min, followed by 29 cycles of 95°C for 30 s, 53°C for 30 s, and 72°C for 45 s, with a final extension at 72°C for 10 min. Amplification was performed using an ABI GeneAmp^®^ 9700 thermal cycler (Applied Biosystems, USA). PCR products were verified using 2% agarose gel electrophoresis and purified using a DNA gel cleanup kit (Yuhua, China). Purified amplicons were quantified using a Qubit 4.0 fluorometer (Thermo Fisher Scientific, USA). Sequencing libraries were constructed using the NEXTFLEX^®^ Rapid DNA-Seq Kit (PerkinElmer, USA) following the manufacturer’s protocol, which includes (1): adapter ligation (2), removal of self-ligated adapters using magnetic beads (3), PCR enrichment of the library, and (4) final purification using magnetic beads.

### 2.9 High-throughput sequencing data analysis

Raw paired-end sequencing reads were quality-filtered using fastp (v0.19.6) (36) (https://github.com/OpenGene/fastp), and merged using FLASH (v1.2.11) (42) (http://www.cbcb.umd.edu/software/flash) following these steps (1): Bases with a quality score < 20 at the ends of the reads were trimmed. A 50 bp sliding window was applied, and bases were trimmed from the end if the average quality score within the window dropped below 20. Reads shorter than 50 bp in length after trimming or containing ambiguous bases (N) were discarded (2); Paired-end reads were merged based on an overlap of ≥10 bp (3); The maximum mismatch rate allowed in the overlap region was 0.2, and sequences not meeting this criterion were discarded (4); Reads were assigned to samples based on barcode and primer sequences at both ends: zero mismatches were allowed for barcodes and up to two mismatches for primers. The processed sequences were clustered into Operational Taxonomic Units (OTUs) using UPARSE v7.1 (43) (http://drive5.com/uparse/) with a 97% similarity threshold. Chimeric sequences, as well as chloroplast and mitochondrial sequences, were removed. To minimize the impact of sequencing depth on downstream alpha and beta diversity analyses, all samples were rarefied to 50,406 sequences. Taxonomic classification of OTUs was performed using the RDP Classifier (v2.11) (44) (http://rdp.cme.msu.edu/) against the Silva 16S rRNA database (v138) with a confidence threshold of 70%. The Shannon diversity index was calculated using mothur (45) (http://www.mothur.org/wiki/Calculators), and inter-group alpha diversity differences were assessed using the Wilcoxon rank-sum test. Principal coordinates analysis (PCoA) based on Bray-Curtis dissimilarity was conducted to assess microbial community structure. PERMANOVA was used to test for significant differences in beta diversity among groups. LEfSe (Linear Discriminant Analysis Effect Size) [(LDA > 2, *p* < 0.05)] was applied to identify differentially abundant bacterial taxa across groups at multiple taxonomic levels.

## 3 Results

### 3.1 Assessing DIV1 susceptible and resistant families of *Macrobrachium rosenbergii*

**Figure 1:**
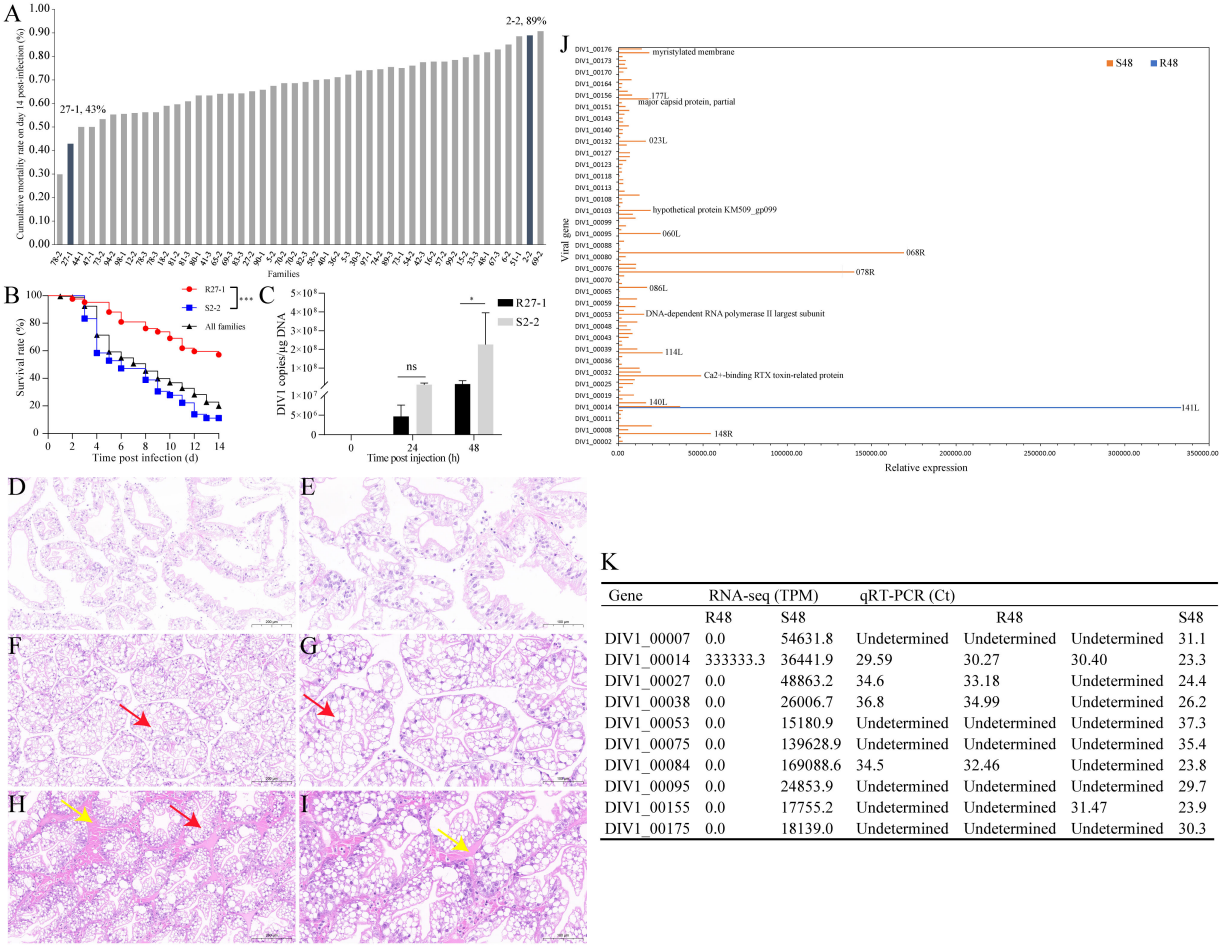
Assessing DIV1 susceptible and resistant families of *M. rosenbergii*. **(A)** Cumulative mortality rates on day 14 post injection of the tested 46 families. **(B)** Survival rate of families R27-1, S2-2, and all experimental families following DIV1 infection. Significant difference between families R27–1 and S2–2 is indicated with *** (Log-Rank test, *p* < 0.0001). **(C)** DIV1 copy numbers in the hepatopancreas from DIV1-infected shrimp of R27–1 and S2–2 families. Data are represented as mean ± S.D. (n = 4). Significant differences between the two families at 24 hpi and 48 hpi are indicated with * (Student’s t-test, *p* < 0.05). ns means no significant difference. **(D–I)** Histopathological examination of H&E-stained hepatopancreas samples from the control group **(D, E)**, the resistant family group **(F, G)**, and the susceptible family group **(H**, **I)** of *M. rosenbergii*. The red arrows indicated cytoplasmic vacuoles, while the yellow arrows indicated eosinophilic material. Scale bars: 200 μm **(D, F, H)** and 100 μm **(E, G, I)**. **(J)** The viral gene sequences detected in the transcriptome data of families R27–1 and S2–2 at 48 hours post-DIV1 infection. **(K)** qRT-PCR validation of 10 key differentially expressed viral transcripts.

**Figure 1A** presents the cumulative mortality rates of the 46 tested families at day 14 post-DIV1 injection. Families 78-2 (30% mortality) and 69-2 (91% mortality) were excluded from further experiments due to limited sample sizes. Instead, family 27-1 (43% mortality) and family 2-2 (89% mortality), which exhibited the second-lowest and second-highest mortality rates, respectively, were selected to represent the resistant (R27-1) and susceptible (S2-2) families for subsequent analyses. **Figure 1B** shows the survival curves of families 27-1, 2-2, and all experimental families following DIV1 infection, revealing a statistically significant difference in mortality between families 27–1 and 2-2 (Log-rank test, *p* < 0.0001). TaqMan probes and primers targeting the *ATPase* gene of DIV1 were used to quantify the viral loads in shrimp muscles. DIV1 was undetectable in the muscles of both uninfected R27–1 and S2–2 lines. At 24 hpi, the viral copy number in S2–2 was approximately 2.4-fold higher than that in R27-1. By 48 hpi, viral loads in S2–2 were significantly elevated (Student’s t-test, *p* < 0.05), reaching levels ~15.7-fold higher than those in R27-1 (**Figure 1C**).

### 3.2 Histopathological observations

As shown in **Figures 1D–I**, the hepatopancreas of the control group exhibited an intact structure with loosely arranged tubules and normal morphology. In comparison to the control group, the hepatopancreatic tubules of the resistant R27–1 family were more compactly arranged, with mild atrophy of the tubule lumen and evident cytoplasmic vacuolization (red arrow). In contrast, the hepatopancreas of the susceptible S2–2 family showed a markedly denser arrangement of tubules, with severe atrophy of the tubule lumen, extensive cytoplasmic vacuolization, and prominent deposition of eosinophilic material in the stroma (yellow arrow). These histopathological differences observed at 48 hours post-DIV1 infection supported the variations in viral loads between the two families, further substantiating the significant differences in their susceptibility to DIV1.

### 3.3 Viral genes detected in transcriptomic data

Previously, we performed whole-genome sequencing of DIV1, resulting in a complete genome assembly and preliminary gene annotation. The assembled genome is 166,964 bp in length, with a GC content of 34.56%, and contains 176 predicted open reading frames (ORFs). To evaluate viral transcriptional activity during infection, we aligned quality-controlled RNA-seq data to the DIV1 genome (GenBank accession number: PQ724921). No viral transcripts were detected in either the resistant or susceptible family groups at 0 and 24 hpi. In contrast, at 48 hpi, only one viral transcript (DIV1_00014) was detected in the resistant group, whereas transcripts corresponding to 103 DIV1-encoded genes were identified in the susceptible group (**Figure 1J**, **Supplementary Table S2**). Although the functions of most viral genes remain uncharacterized, several annotated genes were identified among the detected transcripts, including DNA-dependent RNA polymerase II largest subunit, Ca^2+^-binding RTX toxin-related protein, major capsid protein, partial, and myristylated membrane. To confirm the accuracy of the RNA-seq data, we performed qRT-PCR validation on 10 key differentially expressed viral transcripts, which showed expression trends consistent with the transcriptomic results, further supporting the reliability of our analysis (**Figure 1K**). Primers were shown in **Supplementary Table S1**.

### 3.4 Transcriptome sequencing data

A total of 18 samples were subjected to transcriptome sequencing. Each sequencing library generated between 42,051,634 and 52,033,436 raw reads. Following quality control, 41,699,334 to 51,644,420 clean reads were retained, with each sample yielding over 6.24 Gb of clean data. The proportion of bases with a quality score ≥Q30 exceeded 96% across all samples, indicating high sequencing accuracy (**Supplementary Table S3**), indicating high sequencing quality. Clean reads were subsequently aligned to the *M. rosenbergii* reference genome, with mapping rates ranging from 92.86% to 95.38% (**Supplementary Table S4**). The genomic distribution of mapped reads was analyzed across different genomic features, including coding sequences (CDSs), introns, intergenic regions, and 5’ and 3’ untranslated regions (UTRs). Among all samples, the highest proportion of mapped reads localized to CDS regions (56.93%–70.89%), followed by 3’ UTRs (14.24%–28.10%), 5’ UTRs (8.29%–10.55%), introns (3.51%–5.72%), and intergenic regions (1.35%–3.31%) (**Supplementary Table S5**). Additionally, chromosomal distribution of the mapped reads revealed the highest read densities on chromosomes NC_089753.1, NC_089747.1, and NC_089741.1 (**Supplementary Figure 1**).

### 3.5 Overall transcriptome comparison between the R27–1 and S2–2 families in the absence and presence of infection

**Figure 2:**
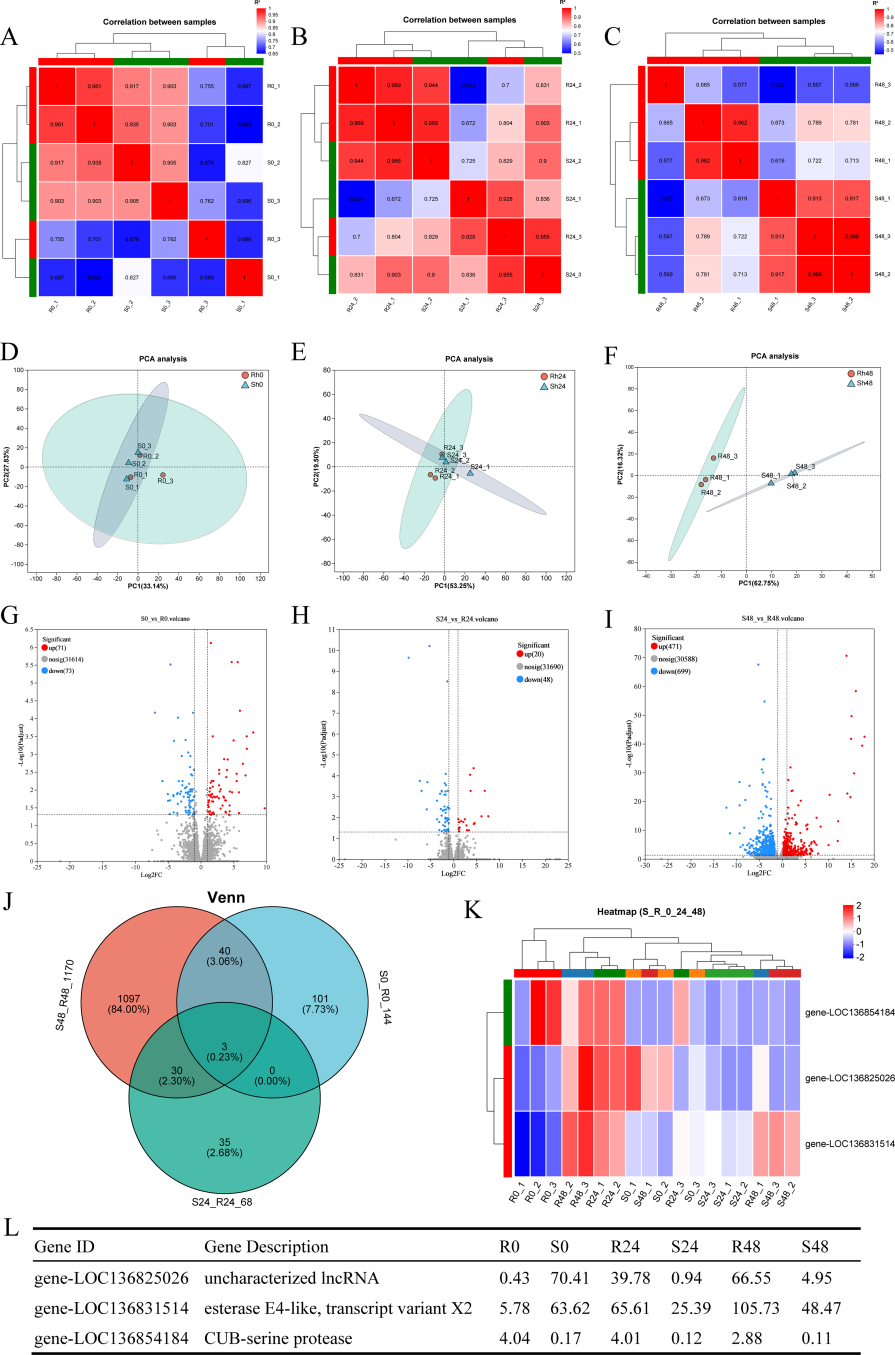
**(A–C)** Correlation heatmap of gene expression among samples from families R27–1 and S2–2 at 0 h, 24 hpi, and 48 hpi. **(D–F)** Principal Component Analysis (PCA) of gene expression among samples from families R27–1 and S2–2 at 0 h, 24 hpi, and 48 hpi. **(G–I)** Volcano plot of differential gene expression comparing S0 vs R0, S24 vs R24, and S48 vs R48. “Up” denotes genes significantly upregulated in the susceptible family S2–2 compared to R27-1, while “Down” indicates genes significantly upregulated in the resistant family R27-1. **(J)** Venn diagram analysis across all three comparison groups: S0 vs R0, S24 vs R24, and S48 vs R48. **(K)** Clustering analysis of the relative expression levels of the 3 shared DEGs across all comparisons. The colors in the graph represent the expression levels of the gene in each sample after normalization, with red indicating higher expression and blue indicating lower expression in that sample. **(L)** Gene expression levels of the 3 shared DEGs in different groups.

Correlation and PCA analyses were performed based on the gene expression matrix to evaluate global transcriptomic differences between the R27–1 and S2–2 families. Correlation analysis assessed the consistency of gene expression among samples, while PCA evaluated overall clustering patterns. As shown in the correlation heatmaps (**Figures 2A–C**) and PCA plots (**Figures 2D–F**), there were no significant differences in gene expression profiles between the R27–1 and S2–2 families at 0 h and 24 hpi. In contrast, at 48 hpi, a marked transcriptomic divergence between the two families was observed, with distinct clustering of samples based on family type. Notably, the resistant S2–2 family exhibited a tighter clustering pattern than the susceptible R27–1 family, indicating a more consistent transcriptional response to DIV1 infection. Differential expression analysis identified 144 (71 upregulated and 73 downregulated), 68 (20 upregulated and 48 downregulated), and 1170 (471 upregulated and 699 downregulated) DEGs in the S2–2 family relative to the R27–1 family at 0 h, 24 hpi, and 48 hpi, respectively (**Figures 2G–I**). The complete lists of DEGs across time points are provided in **Supplementary File S1**. A Venn diagram analysis revealed three DEGs shared across all comparisons (S0 vs R0, S24 vs R24, and S48 vs R48), including an uncharacterized long non-coding RNA (lncRNA), an esterase E4-like protein, and a CUB-serine protease (**Figure 2J**). Clustering heatmap analysis further showed that the relative expression of LOC136854184 (CUB-serine protease) was consistently higher in the resistant S2–2 family compared to the susceptible R27–1 family at all time points (**Figures 2K, L**).

### 3.6 Clustering and KEGG enrichment analyses of the DEGs between the R27–1 and S2–2 families

**Figure 3:**
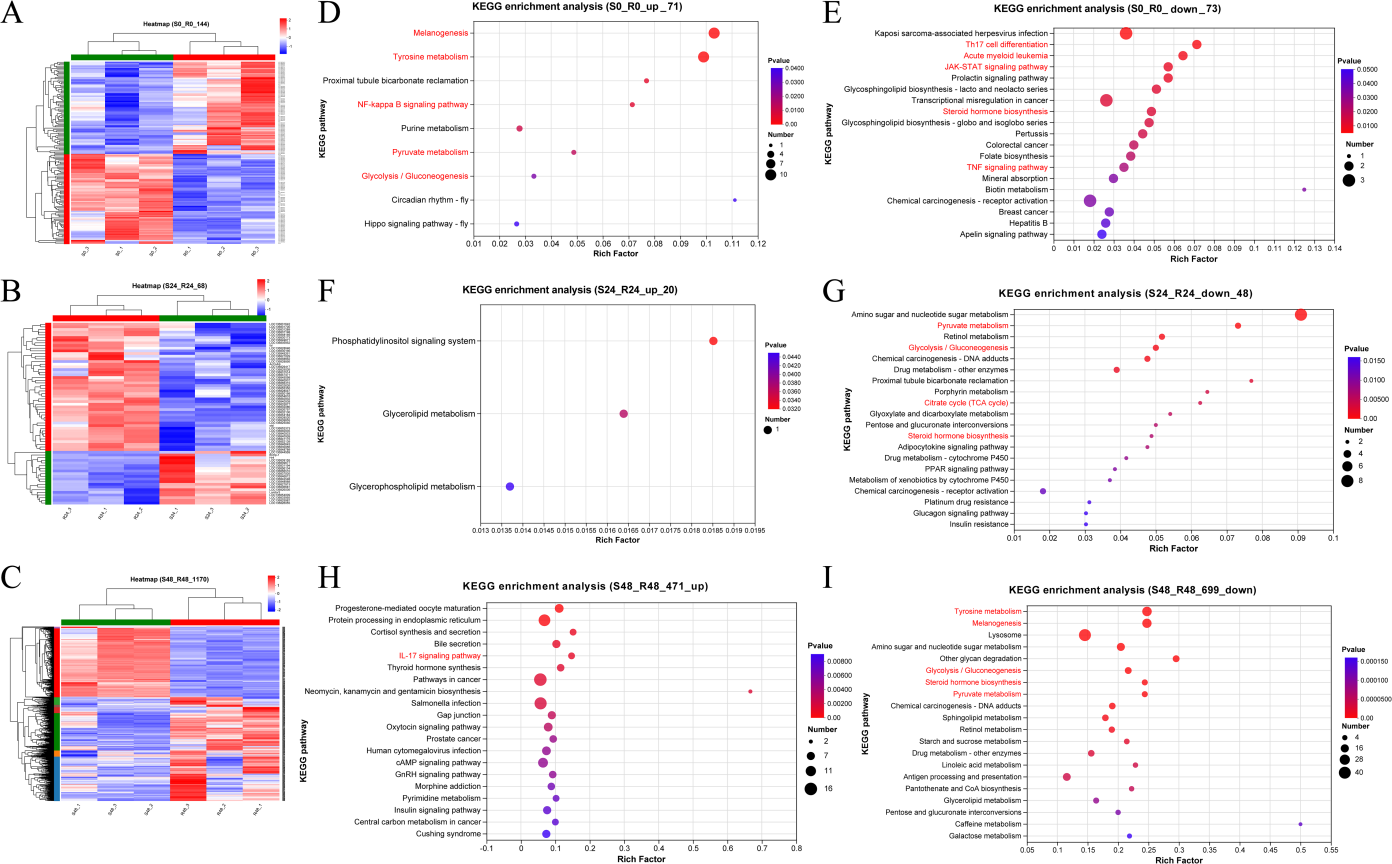
Hierarchical clustering analysis of the relative expression levels of 144 DEGs at 0 h **(A)**, 68 DEGs at 24 hpi **(B)**, and 1170 DEGs at 48 hpi **(C)** from the comparison between S2–2 and R27-1. The gene clustering was performed using the Average linkage method, and the sample clustering used the Complete linkage method, with Euclidean distance as the distance measure for both gene and sample clustering. KEGG functional enrichment analysis was performed on 71 significantly up-regulated DEGs **(D)** and 73 significantly down-regulated DEGs **(E)** at 0 hours; 20 up-regulated DEGs **(F)** and 48 down-regulated DEGs **(G)** at 24 hpi; and 471 up-regulated DEGs **(H)** and 699 down-regulated DEGs **(I)** at 48 hpi, based on the comparison between S2–2 and R27-1. The pathways marked in red represent important or of interest metabolic and immune-related pathways.

Hierarchical clustering was performed on the DEGs at each time point to explore expression differences between the R27–1 and S2–2 families. Gene clustering used the Average method, and sample clustering used the Complete method, with Euclidean distance for both. As shown in **Figures 3A–C**, the 144 DEGs at 0 h, 68 DEGs at 24 hpi, and 1,170 DEGs at 48 hpi were divided into two major clusters, clearly separating the resistant and susceptible families based on their transcriptomic profiles. To further elucidate functional differences, KEGG pathway enrichment analysis was conducted on the DEGs from the S0 vs R0 comparison. Specifically, 71 DEGs highly expressed in the susceptible family and 73 DEGs highly expressed in the resistant family were analyzed (**Figure 3D**). In the susceptible S2–2 family, the enriched pathways included Melanogenesis (10 genes), Tyrosine metabolism (10 genes), NF-kappa B signaling pathway (2 genes), Pyruvate metabolism (2 genes), and Glycolysis/Gluconeogenesis (2 genes). In contrast, DEGs upregulated in the resistant R27–1 family were significantly enriched in immune and endocrine-related pathways, such as Kaposi sarcoma-associated herpesvirus infection (3 genes), Th17 cell differentiation (2 genes), TNF signaling pathway (2 genes), Prolactin signaling pathway (2 genes), Acute myeloid leukemia (2 genes), JAK-STAT signaling pathway (2 genes), and Steroid hormone biosynthesis (2 genes) (**Figure 3E**). A detailed summary of DEGs enriched in these pathways is provided in **Table 1**.

**Table 1:** The DEGs significantly enriched in several pathways under the naïve condition.

Gene and pathway	Gene description	Fold change (S0/R0)
Melanogenesis		
LOC136835700	hemocyanin subunit 1-like protein	112.63
LOC136835772	hemocyanin subunit 1-like protein	13.61
LOC136844192	hemocyanin subunit 1-like protein	13.18
LOC136844193	hemocyanin subunit 1-like protein	14.11
LOC136844566	hemocyanin, partial	62.36
LOC136844570	Hemocyanin	17.07
LOC136844572	Hemocyanin	31.97
LOC136844576	Hemocyanin	14.62
LOC136844580	Hemocyanin	10.83
LOC136844582	Hemocyanin	15.99
Tyrosine metabolism		
LOC136835700	hemocyanin subunit 1-like protein	112.63
LOC136835772	hemocyanin subunit 1-like protein	13.61
LOC136844192	hemocyanin subunit 1-like protein	13.18
LOC136844193	hemocyanin subunit 1-like protein	14.11
LOC136844566	hemocyanin, partial	62.36
LOC136844570	hemocyanin	17.07
LOC136844572	hemocyanin	31.97
LOC136844576	hemocyanin	14.62
LOC136844580	hemocyanin	10.83
LOC136844582	hemocyanin	15.99
NF-kappa B signaling pathway		
LOC136849294	cyclooxygense 2	3.68
tefu	serine-protein kinase ATM-like	2.45
Pyruvate metabolism, Glycolysis/Gluconeogenesis		
LOC136851566	aldehyde dehydrogenase X, mitochondrial	7.40
LOC136830169	phosphoenolpyruvate carboxykinase, cytosolic [GTP]-like	128.27
Kaposi sarcoma-associated herpesvirus infection		
LOC136837207	microtubule-associated proteins 1A/1B light chain 3A-like	0.44
LOC136856412	transcription factor kayak-like	0.29
LOC136840575	myc proto-oncogene protein-like	0.46
Th17 cell differentiation, TNF signaling pathway, Prolactin signaling pathway		
LOC136856412	transcription factor kayak-like	0.29
LOC136850591	interferon regulatory factor 4-like	0.06
Acute myeloid leukemia, JAK-STAT signaling pathway		
LOC136841759	serine/threonine-protein kinase pim-3-like	0.40
LOC136840575	myc proto-oncogene protein-like	0.46
Steroid hormone biosynthesis		
LOC136841405	sulfotransferase 1A1-like	0.11
LOC136842857	cytochrome P450 9e2-like	0.04

At 24 hpi, KEGG enrichment analysis revealed that the 20 DEGs highly expressed in the susceptible family were primarily enriched in the Phosphatidylinositol signaling system (1 gene) (**Figure 3F**). In contrast, the 48 DEGs upregulated in the resistant family were enriched in Amino sugar and nucleotide sugar metabolism (8 genes), Pyruvate metabolism (3 genes), and Glycolysis/Gluconeogenesis (3 genes) (**Figure 3G**). For the S48 vs R48 comparison at 48 hpi, the 471 DEGs highly expressed in the susceptible family were significantly enriched in pathways related to reproduction, protein processing, and hormone response, including Progesterone-mediated oocyte maturation (8 genes), Protein processing in the endoplasmic reticulum (14 genes), and Cortisol synthesis and secretion (5 genes) (**Figure 3H**). Meanwhile, the 699 DEGs highly expressed in the resistant family were predominantly enriched in Tyrosine metabolism (25 genes), Melanogenesis (24 genes), Lysosome (40 genes), and Amino sugar and nucleotide sugar metabolism (18 genes) (**Figure 3I**). A comprehensive summary of DEGs enriched in these pathways is presented in **Table 2**.

**Table 2:** The DEGs in the top 4 significantly enriched pathways of the 699 DEGs at 48 hpi.

Gene name	Log2FC (S48/R48)	Pvalue	NR description
Tyrosine metabolism			
LOC136844567	-4.48	4.97E-72	hemocyanin
LOC136844582	-3.25	1.58E-38	hemocyanin
LOC136844576	-3.45	2.52E-38	hemocyanin
LOC136844580	-3.43	1.02E-33	hemocyanin
LOC136835700	-8.58	2.59E-30	hemocyanin subunit 1-like protein
LOC136844570	-3.52	1.49E-29	hemocyanin
LOC136844673	-3.55	3.34E-29	hemocyanin
LOC136844572	-2.97	1.86E-28	hemocyanin
LOC136844192	-7.31	8.81E-24	hemocyanin subunit 1-like protein
LOC136844581	-3.71	1.94E-17	hemocyanin
LOC136835774	-3.33	2.61E-14	hemocyanin subunit 1-like protein
LOC136835697	-3.42	4.03E-11	hemocyanin subunit 1
LOC136835772	-3.18	1.07E-08	hemocyanin subunit 1-like protein
LOC136844193	-3.14	2.52E-08	hemocyanin subunit 1-like protein
LOC136851253	-1.53	7.68E-07	alcohol dehydrogenase class-3-like
LOC136844579	-5.81	1.07E-06	hemocyanin, partial
LOC136835699	-3.67	1.31E-06	hemocyanin isoform 2
LOC136844577	-2.85	1.52E-06	hemocyanin, partial
LOC136845196	-8.38	2.00E-06	hemocyanin, partial
LOC136835698	-3.53	7.02E-06	hemocyanin subunit 1-like protein
LOC136844566	-3.14	7.63E-06	hemocyanin, partial
LOC136832088	-2.26	4.26E-05	hemocyanin beta subunit 1
LOC136845195	-3.99	5.45E-05	hemocyanin, partial
LOC136852016	-2.23	8.26E-05	aromatic-L-amino-acid decarboxylase-like
LOC136847233	-1.20	5.93E-03	alcohol dehydrogenase class -3, partial
Melanogenesis			
LOC136844567	-4.48	4.97E-72	hemocyanin
LOC136844582	-3.25	1.58E-38	hemocyanin
LOC136844576	-3.45	2.52E-38	hemocyanin
LOC136844580	-3.43	1.02E-33	hemocyanin
LOC136835700	-8.58	2.59E-30	hemocyanin subunit 1-like protein
LOC136844570	-3.52	1.49E-29	hemocyanin
LOC136844673	-3.55	3.34E-29	hemocyanin
LOC136844572	-2.97	1.86E-28	hemocyanin
LOC136844192	-7.31	8.81E-24	hemocyanin subunit 1-like protein
LOC136844581	-3.71	1.94E-17	hemocyanin
LOC136835774	-3.33	2.61E-14	hemocyanin subunit 1-like protein
LOC136835697	-3.42	4.03E-11	hemocyanin subunit 1
LOC136835772	-3.18	1.07E-08	hemocyanin subunit 1-like protein
LOC136844193	-3.14	2.52E-08	hemocyanin subunit 1-like protein
LOC136844579	-5.81	1.07E-06	hemocyanin, partial
LOC136835699	-3.67	1.31E-06	hemocyanin isoform 2
LOC136844577	-2.85	1.52E-06	hemocyanin, partial
LOC136845196	-8.38	2.00E-06	hemocyanin, partial
LOC136835698	-3.53	7.02E-06	hemocyanin subunit 1-like protein
LOC136844566	-3.14	7.63E-06	hemocyanin, partial
LOC136832088	-2.26	4.26E-05	hemocyanin beta subunit 1
LOC136845195	-3.99	5.45E-05	hemocyanin, partial
LOC136844439	-2.79	3.18E-03	frizzled-2-like isoform X1
norpA	-1.17	6.34E-03	1-phosphatidylinositol 4,5-bisphosphate phosphodiesterase-like isoform X2
Lysosome			
LOC136833780	-3.34	2.86E-12	putative inorganic phosphate cotransporter
LOC136848155	-1.40	5.60E-12	alpha-N-acetylgalactosaminidase-like
LOC136835346	-2.50	9.70E-11	legumain-like protein
LOC136847204	-6.17	1.51E-09	putative inorganic phosphate cotransporter isoform X1
LOC136843953	-1.24	6.74E-09	beta-N-acetylglucosaminidase
LOC136833916	-3.46	1.79E-08	putative inorganic phosphate cotransporter
LOC136849838	-1.85	4.96E-08	Flags: Precursor
LOC136845569	-1.43	5.18E-07	putative inorganic phosphate cotransporter
LOC136846903	-4.05	1.97E-06	sialin-like isoform X2
LOC136851518	-1.46	7.29E-06	sialin-like
LOC136850233	-1.82	8.79E-06	beta-mannosidase-like isoform X2
LOC136851881	-1.90	1.13E-05	alpha-L-fucosidase-like isoform X1
LOC136849839	-1.24	1.18E-05	Flags: Precursor
LOC136844805	-1.95	1.52E-05	NPC intracellular cholesterol transporter 2 homolog a-like
LOC136848160	-1.46	1.53E-05	palmitoyl-protein thioesterase 1-like isoform X2
LOC136849127	-1.75	1.65E-05	arylsulfatase A-like
LOC136848855	-2.03	1.82E-05	beta-galactosidase-like
LOC136828129	-1.10	2.34E-05	lysosomal acid glucosylceramidase-like
LOC136856572	-1.44	8.92E-05	glucosylceramidase-like
LOC136831395	-2.05	2.40E-04	cathepsin L, partial
LOC136836348	-1.19	2.47E-04	beta-hexosaminidase subunit alpha-like
LOC136831525	-3.32	2.92E-04	arylsulfatase B-like
LOC136835826	-2.58	3.01E-04	legumain-like
LOC136847721	-1.17	3.09E-04	procathepsin L-like isoform X1
LOC136832166	-1.44	3.43E-04	alpha-L-fucosidase-like
LOC136831383	-1.49	3.58E-04	cathepsin L1
LOC136829434	-1.19	3.65E-04	lysosomal alpha-mannosidase-like isoform X2
LOC136849186	-1.20	3.89E-04	lysosomal alpha-glucosidase-like
LOC136831393	-1.84	4.38E-04	cathepsin L, partial
LOC136831396	-1.40	4.91E-04	cathepsin L, partial
LOC136826203	-1.36	5.16E-04	arylsulfatase B-like
LOC136840726	-1.90	1.28E-03	alpha-N-acetylgalactosaminidase-like
LOC136835828	-2.51	1.36E-03	legumain-like protein
LOC136829657	-2.99	1.42E-03	arylsulfatase B-like
LOC136835564	-1.88	1.71E-03	alpha-L-fucosidase-like
LOC136833362	-1.25	2.14E-03	putative cathepsin L
LOC136831718	-1.09	2.65E-03	cathepsin L, partial
LOC136835323	-1.46	3.61E-03	alpha-N-acetylglucosaminidase-like isoform X3
LOC136829317	-1.39	4.26E-03	sphingomyelin phosphodiesterase-like isoform X2
LOC136845835	-1.14	6.27E-03	cathepsin B
Amino sugar and nucleotide sugar metabolism			
LOC136840933	-1.75	1.72E-09	chitinase 2
LOC136843953	-1.24	6.74E-09	beta-N-acetylglucosaminidase
LOC136840937	-1.72	6.94E-08	chitinase 3C
LOC136841175	-1.58	3.81E-07	chitinase 3C
LOC136852373	-1.80	7.71E-07	chitinase 1C
Nagk	-1.21	1.07E-05	N-acetyl-D-glucosamine kinase-like
LOC136825260	-1.34	1.22E-05	chitinase 4
LOC136825256	-1.14	2.58E-05	chitinase 4
LOC136852371	-1.62	3.05E-05	chitinase 1B
LOC136840935	-1.77	9.76E-05	chitinase 3A
Oscillin	-1.40	1.02E-04	glucosamine-6-phosphate isomerase-like
LOC136836348	-1.19	2.47E-04	beta-hexosaminidase subunit alpha-like
LOC136855846	-3.58	3.55E-04	probable chitinase 2
Uxs	-1.40	7.89E-04	UDP-glucuronic acid decarboxylase 1-like isoform X1
LOC136840939	-1.53	1.80E-03	chitinase
LOC136843696	-1.41	1.96E-03	UTP–glucose-1-phosphate uridylyltransferase-like isoform X1
mmy	-1.52	2.60E-03	UDP-N-acetylhexosamine pyrophosphorylase-like isoform X2
LOC136840938	-1.27	4.13E-03	Chitinase 3

### 3.7 Comparison of the transcriptome profiles between the R27–1 and S2–2 families

KEGG enrichment analysis revealed that transcriptomic differences between the resistant and susceptible families were primarily associated with immune regulation and metabolic pathways (**Figure 3**). In line with these findings, **Table 3** presents the top 20 DEGs that were significantly up- or down-regulated in the S2–2 family compared to the R27–1 family under both uninfected and DIV1-infected conditions, providing a representative overview of the overall transcriptomic variation. Under un-infected conditions, genes encoding alkaline phosphatase-like, Friend leukemia integration 1 transcription factor-like, cytochrome P450 9e2-like, interferon regulatory factor 4-like, dual specificity protein phosphatase 10-like, trypsin II-P29-like, cytochrome c oxidase subunit III, histone H1-delta-like, sodium/calcium exchanger Calx-like, and short coiled-coil protein homolog were highly expressed in the resistant R27–1 family. In contrast, higher expression levels were observed in the susceptible S2–2 family for transcripts including an uncharacterized lncRNA, phosphoenolpyruvate carboxykinase (cytosolic [GTP]-like), hemocyanin subunit 1-like, hemocyanin B chain-like, myosin heavy chain (muscle-like), clotting factor G beta subunit-like, vrille, and mitochondrial D-beta-hydroxybutyrate dehydrogenase-like.

**Table 3:** Top 20 up- and down-regulated DEGs in hepatopancreas samples between the resistant and susceptible families under naïve and infected conditions.

Gene description	Fold change	Gene description	Fold change
Down-0 h (S0/R0)		Up-0 h (S0/R0)	
alkaline phosphatase-like	0.01	uncharacterized lncRNA	254.20
Friend leukemia integration 1 transcription factor-like	0.02	uncharacterized protein	129.30
cytochrome P450 9e2-like	0.04	phosphoenolpyruvate carboxykinase, cytosolic [GTP]-like	128.27
interferon regulatory factor 4-like	0.06	hemocyanin subunit 1-like protein	112.63
uncharacterized protein	0.09	uncharacterized lncRNA	85.47
uncharacterized protein	0.10	hemocyanin B chain-like	62.36
dual specificity protein phosphatase 10-like	0.13	uncharacterized lncRNA	49.15
trypsin II-P29-like	0.14	uncharacterized protein	44.48
uncharacterized protein	0.14	myosin heavy chain, muscle-like	44.22
uncharacterized protein	0.20	hemocyanin B chain-like	31.97
uncharacterized protein	0.22	uncharacterized lncRNA	26.01
cytochrome c oxidase subunit III	0.26	hemocyanin	17.07
histone H1-delta-like	0.29	hemocyanin B chain-like	15.99
sodium/calcium exchanger Calx-like	0.32	hemocyanin	14.62
short coiled-coil protein homolog	0.33	uncharacterized lncRNA	11.74
uncharacterized protein	0.36	clotting factor G beta subunit-like	7.00
RILP-like protein homolog	0.40	uncharacterized protein	6.92
synaptic vesicle glycoprotein 2B-like	0.44	vrille	3.54
ras-related protein ced-10-like	0.48	uncharacterized protein	3.22
uncharacterized protein	0.48	D-beta-hydroxybutyrate dehydrogenase, mitochondrial-like	2.90
Down-24 hpi (S24/R24)		Up-24 hpi (S24/R24)	
uncharacterized lncRNA	0.00	hemocyanin B chain-like	191.92
sodium-coupled monocarboxylate transporter 1-like	0.01	peritrophin-1-like	112.05
uncharacterized lncRNA	0.01	uncharacterized lncRNA	68.68
uncharacterized lncRNA	0.02	nitrate reductase [NADH] 1-like	24.29
astacin-like metalloendopeptidase	0.03	Hormone receptor 4	21.47
thiol S-methyltransferase TMT1A-like	0.08	solute carrier family 15 member 2-like	20.77
organic cation transporter protein-like	0.13	nose resistant to fluoxetine protein 6-like	13.24
uncharacterized protein	0.17	uncharacterized protein	12.23
glutathione S-transferase 1-like	0.17	PR domain zinc finger protein 1	10.23
chitinase-3-like protein 1	0.25	juvenile hormone esterase-like	7.64
phosphoenolpyruvate carboxykinase, cytosolic [GTP]-like	0.25	uncharacterized protein	7.06
tripartite motif containing 13-like	0.27	transmembrane protein 135-like	5.59
multidrug resistance-associated protein 1-like	0.29	carbohydrate sulfotransferase 1-like	4.40
acidic mammalian chitinase-like	0.30	uncharacterized protein	3.13
uncharacterized protein	0.31	eye-specific diacylglycerol kinase-like	2.54
putative aldolase class 2 protein PA3430	0.32	protein Star-like	2.35
falten	0.39	uncharacterized protein	2.33
C-type lectin domain family 17, member A-like	0.41	uncharacterized lncRNA	2.30
glycine dehydrogenase (decarboxylating), mitochondrial	0.42	zinc finger protein 235-like	2.20
transmembrane protein 17B-like	0.48	Late endosomal/lysosomal adaptor, MAPK and MTOR activator 3	2.07
Down-48 hpi (S48/R48)		Up-48 hpi (S48/R48)	
hemocyanin subunit 1-like	0.00	vitellogenin-like	425582.97
organic cation transporter protein-like	0.00	vitellogenin 1b	297646.90
hemocyanin subunit-like	0.01	vitellogenin 1b	114218.82
pancreatic lipase-related protein 2-like	0.01	vitellogenin-like	88000.12
hemocyanin B chain-like	0.05	vitellogenin 1b	59896.38
UDP-glycosyltransferase UGT5-like	0.08	vitellogenin-like	55737.09
hemocyanin-like	0.09	vitellogenin 1b	50157.61
hemocyanin	0.09	vitellogenin-like	31627.58
hemocyanin	0.09	vitellogenin-like	27737.20
hemocyanin B chain-like	0.09	vitellogenin-like	2178.13
hemocyanin B chain-like	0.11	ras guanine nucleotide exchange factor E-like	302.62
phosphoenolpyruvate carboxykinase, cytosolic [GTP]-like	0.11	Y+L amino acid transporter 2-like	27.75
hemocyanin B chain-like	0.13	hexokinase-2-like	19.02
repressed by TOR	0.13	uncharacterized lncRNA	11.34
vitelline membrane outer layer protein 1 homolog	0.14	elongation of very long chain fatty acids protein baldspot	7.96
cysteine dioxygenase-like	0.16	acyl-CoA Delta-9 desaturase-like	6.87
cytochrome P450 9e2-like	0.16	DNA repair protein RAD51 homolog 2-like	5.79
uncharacterized protein	0.18	metalloreductase STEAP4-like	4.18
lethal (1) G0469	0.24	uncharacterized protein	4.15
uncharacterized protein	0.31	bromodomain-containing protein 4B-like	3.82

**Figure 4:**
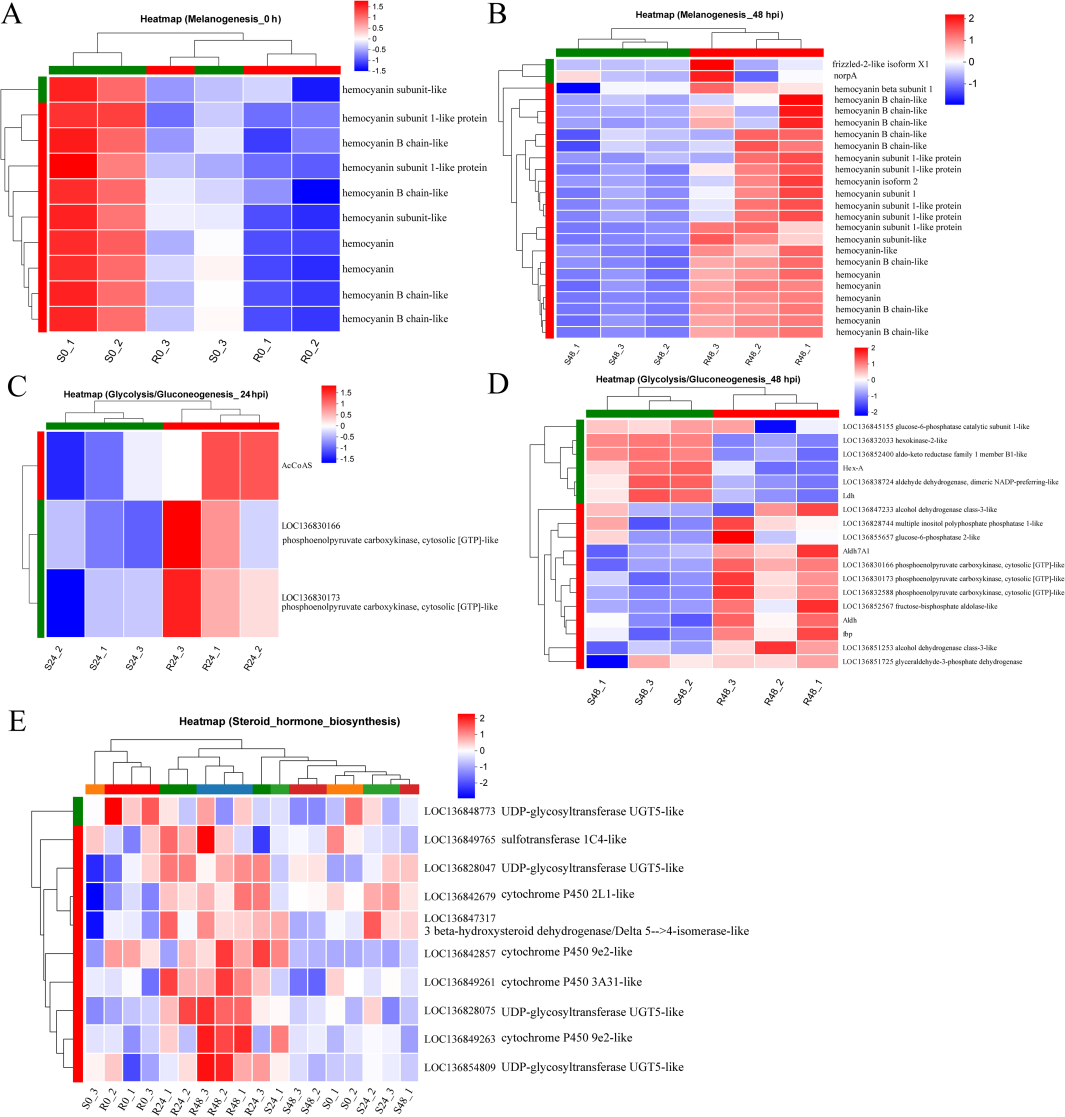
Heatmap of the relative expression levels of DEGs at 0 h **(A)** and 48 hpi **(B)** mapped to the Melanogenesis pathway. Heatmap of the relative expression levels of DEGs at 24 hpi **(C)** and 48 hpi **(D)** mapped to the Glycolysis/Gluconeogenesis. **(E)** Heatmap of the relative expression levels of DEGs mapped to Steroid hormone biosynthesis at 48 hpi across all samples.

The differential expression of the Melanogenesis pathway between the two families prompted further analysis. Interestingly, in the uninfected state, the resistant family R27–1 exhibited lower expression of several genes within this pathway compared to the susceptible family S2-2 (**Figure 4A**). However, by 48 hours post-DIV1 infection, the expression levels of these genes were higher in the resistant family (**Figure 4B**), including genes encoding hemocyanin subunits, frizzled-2-like isoform X1, and norpA. In addition, in the uninfected state, two DEGs—aldehyde dehydrogenase X and phosphoenolpyruvate carboxykinase—were highly expressed in the susceptible family and enriched in the Glycolysis/Gluconeogenesis pathway. At 24 hpi and 48 hpi, 3 (**Figure 4C**) and 12 DEGs (**Figure 4D**), respectively, were highly expressed in the resistant family and similarly enriched in this pathway.

Another notable pathway was Steroid hormone biosynthesis, which was significantly enriched in the DEGs highly expressed in the resistant family across both uninfected and infected states (**Figures 3E, G, I**). This pathway is intimately linked to lipid metabolism and is essential for the synthesis of steroid hormones that regulate a wide array of physiological processes. The DEGs involved in this pathway include: 3 beta-hydroxysteroid dehydrogenase/Delta 5–>4-isomerase-like, Cytochrome P450 9e2-like, Cytochrome P450 2L1-like, UDP-glycosyltransferase UGT5-like, UDP-glycosyltransferase UGT5-like, Sulfotransferase 1C4-like, UDP-glycosyltransferase UGT5-like, Cytochrome P450 9e2-like, Cytochrome P450 3A31-like, UDP-glycosyltransferase UGT5-like (**Figure 4E**).

### 3.8 Differences in intestinal microbial community composition between R27–1 and S2–2 families

**Figure 5:**
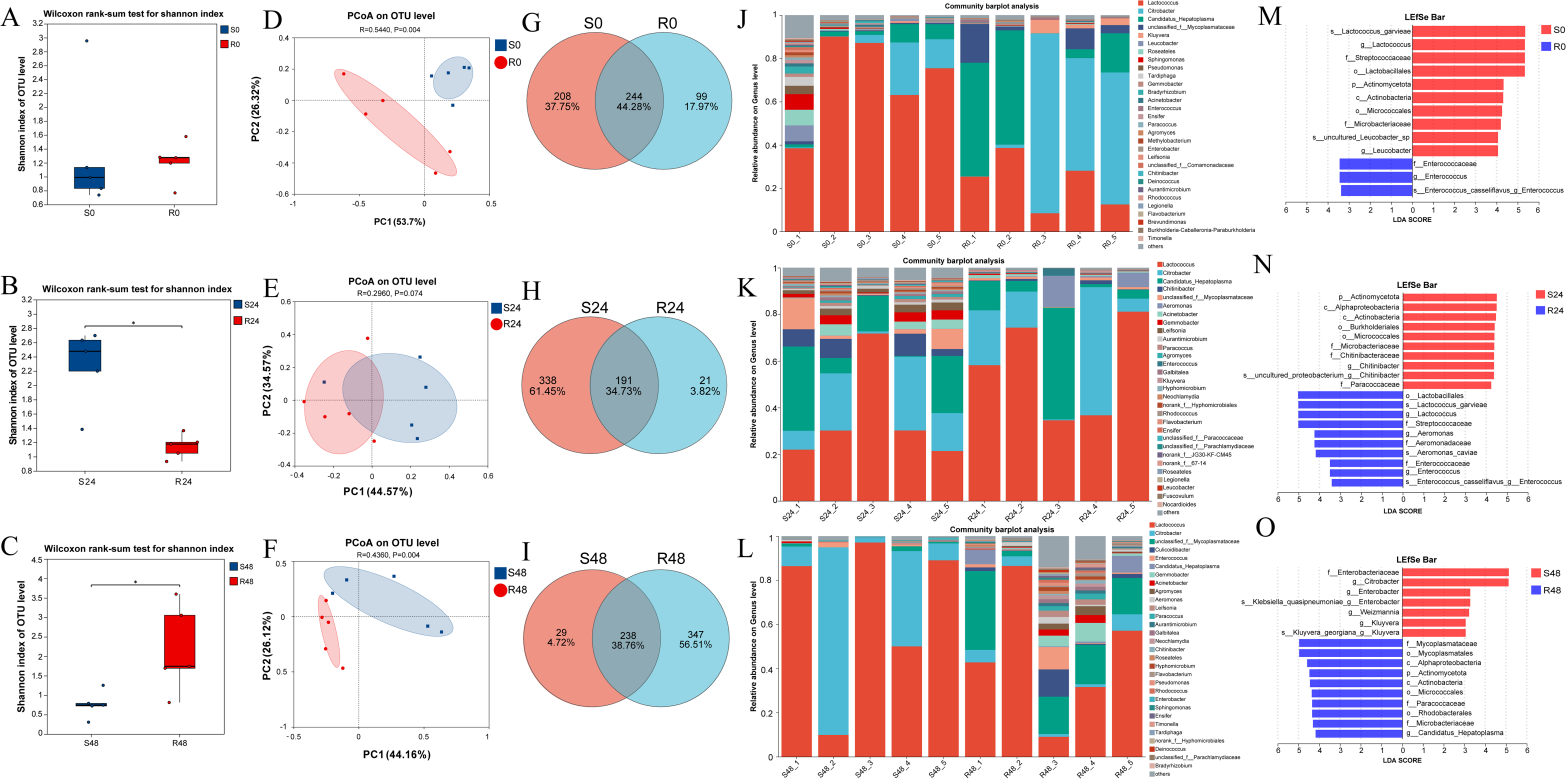
Shannon index-based microbial diversity for S0 vs R0 **(A)**, S24 vs R24 **(B)**, and S48 vs R48 **(C)**. Wilcoxon test was used to detect the variation between families R27–1 and S2-2. “*”: *p* < 0.05. **(D–F)** Principal Coordinates Analysis (PCoA) at the OTU level was performed to compare the gut microbiome composition between the susceptible and resistant families at three time points: S0 vs. R0, S24 vs. R24, and S48 vs. R48. **(G–I)** Venn plot at the OTU level for S0 vs R0, S24 vs R24, and S48 vs R48. **(J–L)** Bar plot of community composition showing the relative abundance of dominant taxa at the genus level across all samples and the proportion of different taxa at 0 h, 24 hpi, and 48 hpi. **(M–O)** Lefse multilevel species differential analysis. The bar plot displays the LDA scores of differential species in resistant and susceptible families at 0 h **(M)**, 24 hpi **(N)**, and 48 hpi **(O)**, visually showing the impact of marker taxa on the differential effects. The LDA bar plot statistically identifies microbial taxa with significant effects, with higher LDA scores indicating a greater influence of species abundance on the differential effects.

Shannon diversity indices and Wilcoxon rank-sum tests were used to evaluate gut microbiota diversity between the resistant and susceptible families. At 0 h, no significant difference was observed between the two groups (S0: 1.22; R0: 1.33; **Figure 5A**). However, at 24 hpi, the Shannon index of the susceptible group (S24: 2.27) was significantly higher than that of the resistant group (R24: 1.14), and also notably increased compared to S0. In contrast, the diversity of the resistant family remained relatively stable from R0 to R24 (**Figure 5B**). At 48 hpi, the resistant family exhibited significantly higher diversity (R48: 2.18) than the susceptible group (S48: 0.76; **Figure 5C**). These results were further supported by the Chao and Simpson indices, as shown in **Supplementary Figure 2**. Principal coordinate analysis (PCoA) further revealed marked differences in community composition between the two families, with PC1 and PC2 explaining 80.02%, 79.14%, and 70.28% of the variation at 0, 24, and 48 hpi, respectively (**Figures 5D–F**).

The intestinal microbial communities of the resistant and susceptible families showed distinct differences at the OTU level. At 0 h, a total of 244 OTUs (44.28% of all identified OTUs) were shared between the two families, with 99 OTUs (17.97%) unique to the R27–1 family and 208 OTUs (37.75%) unique to the S2–2 family (**Figure 5G**). At 24 hpi, 191 OTUs (34.73%) were shared, with 21 OTUs (3.82%) specific to the resistant family and 338 OTUs (61.45%) specific to the susceptible family (**Figure 5H**). By 48 hpi, 238 OTUs (38.76%) were shared, whereas 347 OTUs (56.51%) were unique to the resistant family and only 29 OTUs (4.72%) to the susceptible family (**Figure 5I**).

Regarding the relative abundance (RA) of specific taxa at the genus level, *Citrobacter* (RA: 39.55% in the resistant group vs. 8.38% in the susceptible group), *Candidatus Hepatoplasma* (RA: 25.51% vs. 4.15%), unclassified f:*Mycoplasmataceae* (RA: 6.62% vs. 0.66%), *Kluyvera* (RA: 2.20% vs. 0.23%), and *Enterococcus* (RA: 0.51% vs. 0.03%) were more abundant in the resistant family. In contrast, *Lactococcus* (RA: 22.63% vs. 70.88%), *Leucobacter* (RA: 0.003% vs. 1.86%), *Roseateles* (RA: 0.15% vs. 1.63%), *Sphingomonas* (RA: 0.13% vs. 1.58%), and *Pseudomonas* (RA: 0.10% vs. 0.91%) were more abundant in the susceptible family at 0 hours (**Figure 5J**). At 24 hpi, *Lactococcus* (RA: 57.01% vs. 35.01%), *Citrobacter* (RA: 19.98% vs. 12.31%), *Candidatus Hepatoplasma* (RA: 14.04% vs. 16.53%), and *Aeromonas* (RA: 4.08% vs. 0.04%) were more abundant in the resistant family. In contrast, *Chitinibacter* (RA: 0.57% vs. 5.70%), unclassified f:*Mycoplasmataceae* (RA: 0.88% vs. 5.11%), *Acinetobacter* (RA: 0.71% vs. 2.53%), *Gemmobacter* (RA: 0.29% vs. 2.77%), and *Leifsonia* (RA: 0.01% vs. 2.53%) were more abundant in the susceptible family (**Figure 5K**). At 48 hpi, unclassified f:*Mycoplasmataceae* (RA: 17.87% vs. 0.84%), *Culicoidibacter* (RA: 3.28% vs. 0.00%), *Enterococcus* (RA: 2.61% vs. 0.64%), *Candidatus Hepatoplasma* (RA: 3.06% vs. 0.09%), *Gemmobacter* (RA: 3.05% vs. 0.05%), *Acinetobacter* (RA: 1.47% vs. 0.23%), and *Agromyces* (RA: 1.54% vs. 0.02%) were more abundant in the resistant family. On the other hand, *Lactococcus* (RA: 45.42% vs. 66.52%) and *Citrobacter* (RA: 4.02% vs. 29.36%) were more abundant in the susceptible family (**Figure 5L**). The relative abundance data of differential microorganisms at different sampling points are provided in **Supplementary File S2**. LEfSe multi-level differential analysis was performed to assess species-level differences across taxonomic hierarchies. The taxa contributing most significantly to these differences were identified using linear discriminant analysis (LDA), which revealed that s:*Lactococcus garvieae*, g:*Lactococcus*, f:*Streptococcaceae*, and o:*Lactobacillales* in S0; f:*Enterococcaceae*, g:*Enterococcus*, and s:*Enterococcus casseliflavus*:g:*Enterococcus* in R0 (**Figure 5M**); p:*Actinomycetota*, c:*Alphaproteobacteria*, c:*Actinobacteria*, and o:*Burkholderiales* in S24; o:*Lactobacillales*, s:*Lactococcus garvieae*, g:*Lactococcus*, and f:*Streptococcaceae* in R24 (**Figure 5N**); f:*Enterobacteriaceae* and g:*Citrobacter* in S48; and f:*Mycoplasmataceae* and o:*Mycoplasmatales* in R48 had the greatest impact on the observed differences (**Figure 5O**). These results suggest that these taxa may play a crucial role in distinguishing the resistance to DIV1 in *M. rosenbergii*.

### 3.9 Associations between host DEGs and intestinal microbes

**Figure 6:**
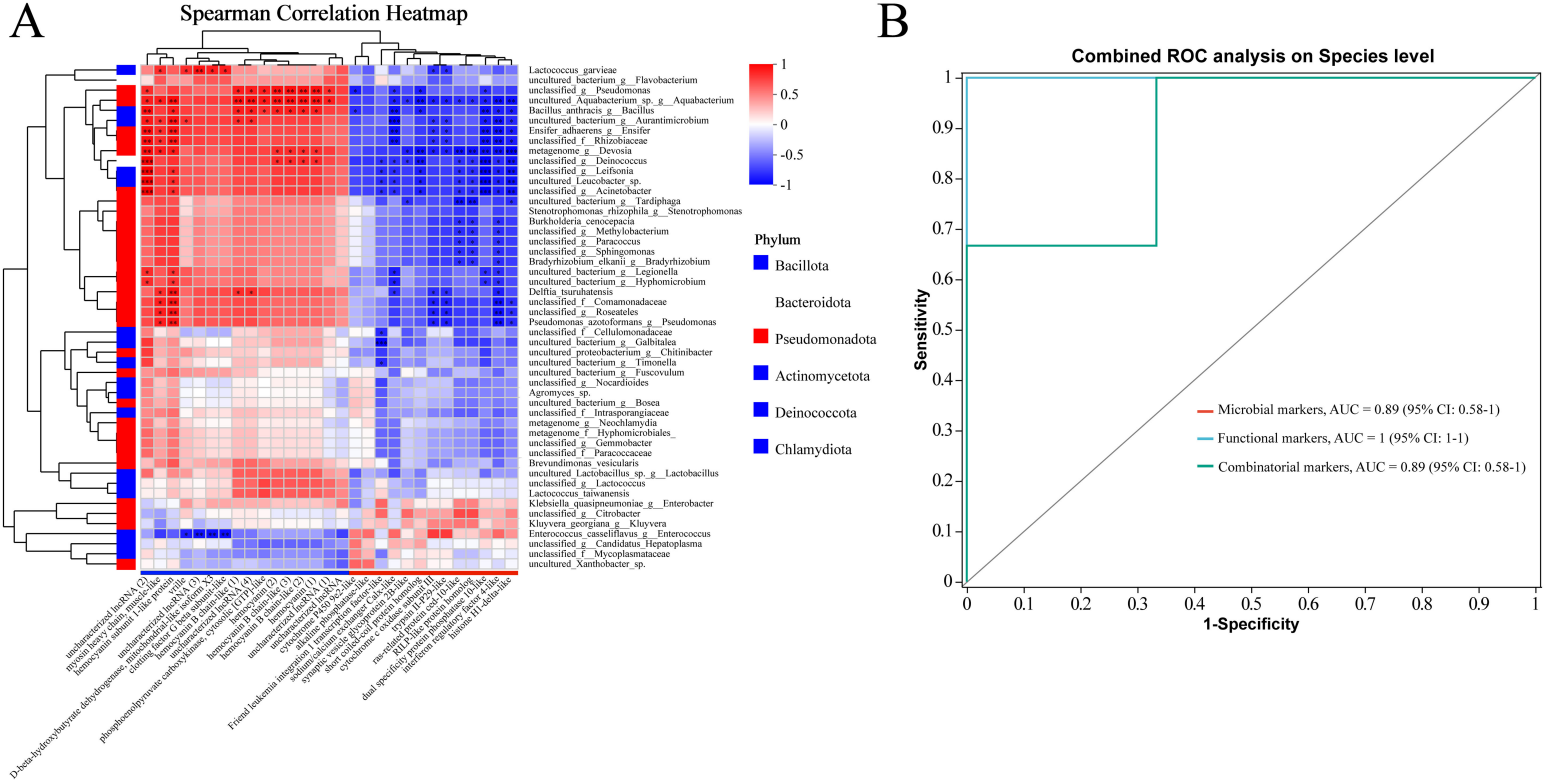
**(A)** The host DEGs (including 13 upregulated and 16 downregulated genes in the resistant family) exhibit family-specific associations with the intestinal microbiome of *M. rosenbergii*. A correlation analysis was performed between 29 DEGs (|log2(FoldChange)| > 1.00, adjusted *p* < 0.05) and the top 50 most abundant species. The results are presented in terms of R and *p* values, with R values represented by a color gradient. *p* values less than 0.05, 0.01, and 0.001 are indicated by *, **, and ***, respectively. In the heatmap, red and blue cells represent positive and negative correlations, respectively. **(B)** Various markers for discriminating the resistant family from the susceptible family in *M. rosenbergii*. Comparison of area under receiver operating characteristic curves (AUC), microbial markers (red curve), functional markers (blue curve), and combined markers (green curve).

To investigate the relationships between host gene expression, the intestinal microbiome, and their potential roles in DIV1 resistance, we explored the associations between 29 DEGs (|log2(FoldChange)| > 1.00, adjusted *p* < 0.05) and the top 50 most abundant species in the absence of infection. We found resistant and susceptible family specific host-microbiome associations (|Spearman rank correlation| ≥ 0.5). In the **Figure 6A**, “*” denotes *p* < 0.05; “**” denotes *p* < 0.01; and “***” denotes *p* < 0.001. Interestingly, most microbes associated with the up- and down-expressed DEGs in the resistant family were also different (**Figure 6A**). That is, 19 microbes (*Lactococcus_garvieae*, *uncultured_bacterium_g:Flavobacterium*, *unclassified_g:Pseudomonas*, *uncultured_Aquabacterium_sp._g:Aquabacterium*, *Bacillus_anthracis_g:Bacillus*, *uncultured_bacterium_g:Aurantimicrobium*, *Ensifer_adhaerens_g:Ensifer*, *unclassified_f:Rhizobiaceae*, *metagenome_g:Devosia*, *unclassified_g:Deinococcus*, *unclassified_g:Leifsonia*, *uncultured_Leucobacter_sp.*, *unclassified_g:Acinetobacter, uncultured_bacterium_g:Legionella*, *uncultured_bacterium_g:Hyphomicrobium*, *Delftia_tsuruhatensis*, *unclassified_f:Comamonadaceae*, *unclassified_g:Roseateles*, *Pseudomonas_azotoformans_g:Pseudomonas*) had negative associations with most up-expressed genes, but were positively associated with the down-expressed genes in the resistant family (**Figure 6A**). On the contrary, four microbes (*Klebsiella_quasipneumoniae_g:Enterobacter*, *unclassified_g:Citrobacter*, *Kluyvera_georgiana_g:Kluyvera*, and *Enterococcus_casseliflavus*) were positively associated with the up-expressed genes, but were negatively associated with the down-expressed genes in the resistant family (**Figure 6A**). These results suggest that these intestinal microbes might interact with the DEGs to modulate DIV1 resistance.

### 3.10 Integrated intestinal microbial and host functional markers for distinguishing resistant from susceptible families

Combinatorial markers distinguished the resistant from the susceptible family. This study assessed the role of intestinal microbes and host functional genes in this differentiation. We found that four microbial markers: *Bacillus anthracis*, *Lactococcus garvieae*, *uncultured_Aquabacterium*, and *Enterococcus casseliflavus* could accurately discriminate the resistant family from the susceptible family (area under curve (AUC): 89%, confidence interval (CI): 0.58–1; red curve; **Figure 6B**). As for functional markers, three host genes associated with uncharacterized lncRNA, an esterase E4-like protein, and a CUB-serine protease (**Figure 2K**) exhibited strong performance (AUC: 100%, CI: 1–1; blue curve; **Figure 6B**). These results suggested the host intestinal gene functional profiles have larger differences between the susceptible and resistant families, compared to the microbial community. We then combined these microbial and functional markers, and their combination provided 89% prediction power in discriminating the resistant family from the susceptible family (AUC: 89%, CI: 0.58–1; green curve; **Figure 6B**).

## 4 Discussion

Selective breeding of disease-resistant shrimp is an effective, practical, and sustainable method for disease control. In this study, we conducted systematic family selection for resistance traits to DIV1 in *M. rosenbergii*.

Based on the differences in survival rate, we identified the susceptible family S2–2 and resistant family R27-1, and then compared the viral load and histopathological changes between these two families. At 24 and 48 hpi, higher DIV1 loads were detected in the hepatopancreas of the susceptible family, suggesting that viral invasion occurs more readily in this group compared to the resistant family. Histopathological analysis also revealed more severe tissue damage in hepatopancreas samples from the susceptible family compared to the resistant family. Furthermore, the discrepancies in viral gene sequences identified from the hepatopancreas transcriptomic data of the resistant and susceptible families provide additional evidence of their differential susceptibility to DIV1. Specifically, at 48 hpi, a substantial number of viral gene sequences were detected in the susceptible family (S2-2), suggesting rapid viral replication and proliferation. In contrast, only the viral sequence 141L was detected in the resistant family (R27-1), indicating that these families possess effective immune responses or antiviral mechanisms that suppress viral replication. Several genes associated with early viral replication—including 068R, 078R, 148R, 141L, DNA-dependent RNA polymerase II largest subunit, and Ca²^+^-binding RTX toxin-related protein—were highly expressed in the susceptible family, highlighting their potential roles in the early phase of infection. DNA-dependent RNA polymerase II is a key enzyme in viral transcription, essential for RNA synthesis, replication, and protein production. RTX toxins, which are involved in cellular invasion and toxicity, likely facilitate viral infection and spread by modulating calcium signaling pathways in host cells (46–48). The high expression of these genes may contribute to rapid replication in the susceptible family, whose host cells may be more vulnerable to invasion and lack sufficient antiviral responses. In contrast, the resistant family displayed lower expression levels of viral genes, possibly due to more robust innate immune responses that rapidly detect and eliminate the virus, thus restricting viral spread, reducing replication, and minimizing viral load. In summary, the susceptible family S2–2 and the resistant family R27–1 serve as valuable models for studying DIV1 resistance in *M. rosenbergii*. To further explore the underlying mechanisms, we conducted RNA sequencing to compare the hepatopancreas transcriptomes and microbiome sequencing to analyze gut microbial composition in both families. To our knowledge, this is the first study to integrate gene expression profiles, gut microbiome, and DIV1 resistance phenotypes in *M. rosenbergii*.

In the hepatopancreas, the Melanogenesis and Tyrosine metabolism pathways exhibit significant differential regulation both in the uninfected and infected states. Notably, under naïve conditions, the resistant family showed lower expression levels of several key genes involved in these pathways, whereas the expression pattern reversed in infected shrimp at 48 hpi. Melanogenesis plays a crucial role in the innate immune response of arthropods and other invertebrates. During this process, toxic quinone compounds and other reactive intermediates are produced, contributing to melanin formation that physically encapsulates and neutralizes invading pathogens (49). Melanocytes are key mediators of this defense, initiating responses to microbial infections. Innate immune stimulation, particularly through the activation of Toll-like receptors (TLRs), enhances melanogenesis and promotes melanin transport, thereby strengthening the elimination of pathogens (50). In this study, the enrichment of melanogenesis pathways observed in the resistant lineage at 48 hpi likely reflects an enhanced capacity for melanin synthesis and distribution, reinforcing both physical and chemical defenses against external pathogens. Therefore, upregulation of Melanogenesis may not only contribute to the heightened immune response observed in the resistant lineage but also represent a critical biological basis for its superior resistance phenotype. Further analysis revealed several highly differentially expressed genes (DEGs) in the Melanogenesis and Tyrosine metabolism pathways closely associated with hemocyanin (HMC). HMC, a large copper-containing respiratory protein in mollusks and arthropods, was long considered solely an oxygen carrier until the late 1990s, when its phenoloxidase (PO) activity was confirmed, revealing its role in innate immunity (51–53). In shrimp, humoral immunity, including the prophenoloxidase (proPO) system, coagulation cascades, and antimicrobial peptides (AMPs), plays a vital role in pathogen defense (54, 55). Key immune molecules include lysozyme, lectins, PO, AMPs, and PmAV (56, 57). Importantly, HMC acquires monophenol and o-diphenol oxidase activities via proteolysis, promoting melanin production and antimicrobial responses (51, 58). It also modulates PO activity and enhances pathogen-induced immune reactions. HMC levels, PO activity, and bacteriolysis peak at 24 hpi following *Staphylococcus aureus* or *Vibrio harveyi* infection (59). Hemocyanin subunits have been shown to inhibit viral replication, block herpesvirus entry, and delay WSSV infection (60, 61). Furthermore, its C-terminal peptides exhibit strong agglutination against pathogens such as *Escherichia coli*, *V. parahaemolyticus*, *Vibrio vulnificus*, and *S. aureus* (62, 63). Together, these findings support the central role of HMC in crustacean immunity. In this study, higher basal HMC expression in the susceptible lineage may reflect elevated metabolic or immune demands under normal conditions, whereas the more pronounced induction observed in the resistant family following DIV1 infection suggests a crucial role for HMC in antiviral defense in *M. rosenbergii*.

Glycolysis and Gluconeogenesis are key metabolic pathways involved in glucose regulation. In an uninfected state, DEGs encoding aldehyde dehydrogenase X (LOC136851566) and phosphoenolpyruvate carboxykinase, cytosolic [GTP]-like (LOC136830169) were highly expressed in S2–2 family compared to R27–1 family. However, at 24 hpi (**Figure 4C**) and 48 hpi (**Figure 4D**), a total of 3 and 12 DEGs, respectively, were significantly upregulated in the resistant family and were markedly enriched in this pathway. This suggests that, in the absence of infection, the S2–2 family may have a higher basal energy demand. Upon DIV1 infection, however, the resistant R27–1 family shows significantly increased expression of glucose metabolism-related genes, likely to meet elevated energy requirements and support immune responses during viral infection. Interestingly, this regulatory pattern is consistent with that observed in the Melanogenesis pathway and may be related to DIV1 resistance. Studies have demonstrated that the activation of steroid hormone signaling plays a crucial role in immune responses and survival following pathogen challenge in *Drosophila* (64). Similarly, a comparative study on resistance and susceptibility in *Cynoglossus semilaevis* to *Vibrio* revealed that DEGs highly expressed in the resistant family were significantly enriched in steroid and bile acid metabolism pathways (23). Our findings align with these results. In the uninfected state, genes involved in the Steroid hormone biosynthesis pathway, including *cytochrome P450 3A31-like* and *UDP-glycosyltransferase UGT5-like*, were highly expressed in the resistant family. Moreover, at 48 hpi, DEGs associated with this pathway, such as *3 beta-hydroxysteroid dehydrogenase/Delta 5–>4-isomerase-like*, *cytochrome P450 9e2-like*, *cytochrome P450 2L1-like*, *UDP-glycosyltransferase UGT5-like*, *sulfotransferase 1C4-like*, and *cytochrome P450 3A31-like*, were also highly expressed in the resistant family (*p* < 0.05, **Figure 4E**). Cytochrome P450 (CYP) enzymes are membrane-associated hemoproteins essential for detoxifying xenobiotics, cellular metabolism, and homeostasis (65). Genetic variations and epigenetic modifications in CYP genes could contribute to differences in disease susceptibility and drug response among individuals and across ethnic groups (65). These genes may serve as potential molecular markers for distinguishing DIV1 resistance, and further validation is needed in future stugies.

In this study, several immune-related genes exhibited significantly higher expression levels in the resistant family compared to the susceptible family, suggesting that they may play key roles in DIV1 resistance. Among them, alkaline phosphatase (AP) showed the most prominent differential expression, indicating a potentially central role in the antiviral immune response. AP functions in anti-inflammatory and detoxification through dephosphorylation, helping to mitigate inflammatory responses and neutralize endotoxins such as lipopolysaccharides (LPS), thereby contributing to tissue homeostasis and protecting organ function under stress conditions such as viral infection (66). For instance, previous studies have demonstrated that AP can hydrolyze ATP into adenosine, which exerts anti-inflammatory and tissue-protective effects, particularly in the kidney and intestinal tract (66). Notably, intestinal alkaline phosphatase (IAP) plays an essential role in maintaining gut homeostasis by detoxifying LPS, enhancing barrier function, and inducing autophagy to clear damaged organelles (67). Additionally, immune regulatory factors such as Friend leukemia integration 1 transcription factor-like (FLI1-like), interferon regulatory factor 4-like (IRF4-like), dual specificity protein phosphatase 10-like (DUSP10-like), and Trypsin II-P29-like also displayed significant expression differences between the two families, highlighting their potential involvement in the immune defense against DIV1. Among these, DUSP10 is a dual-specificity phosphatase that negatively regulates the p38 MAPK and JNK signaling pathways via dephosphorylation, thereby suppressing excessive inflammatory responses and participating in both innate and adaptive immune regulation (68). It is considered a promising anti-inflammatory target. In contrast, Trypsin II-P29-like may contribute to the activation of protease cascades and promote inflammatory responses (69), suggesting that moderate activation of inflammatory pathways may serve as an early immune defense strategy in the resistant family. Supporting this, trypsin expression has also been reported to be higher in AHPND-tolerant *L. vannamei* compared to susceptible individuals (18). FLI1-like, a member of the Ets family of transcription factors, is involved in regulating genes crucial for cell proliferation, differentiation, and apoptosis, and may play a role in hematopoiesis and host defense (70). IRF4 is a key transcriptional regulator that plays a pivotal role in modulating interferon (IFN)-mediated signaling cascades. It has been implicated in multiple immunological processes, including antiviral responses, T helper (Th) cell lineage commitment, and B cell maturation, primarily through the transcriptional regulation of IFNs and various lymphokines involved in immune cell differentiation and function (71). Taken together, these DEGs not only reveal potential molecular mechanisms underlying the resistant family’s response to DIV1 infection, but also provide a theoretical foundation and candidate targets for further functional validation, marker-assisted selection, and disease-resistant breeding.

Commensal microorganisms and their eukaryotic hosts have evolved together over time, forming a complex and mutually advantageous relationship (72). Although there was no significant difference in the Shannon index of the gut microbiota between the resistant and susceptible lines uninfected with DIV1, the index was higher in the resistant line, which is consistent with previous studies showing that the mid-gut bacterial community of the uninfected susceptible rainbow trout exhibited significantly greater diversity than that of the resistant line (72). In addition, based on both the diversity index and community composition results, the resistant family exhibited a greater capacity to maintain microbiome stability than the susceptible family. *L. garvieae* is a potential pathogen responsible for considerable economic losses in both marine and freshwater aquaculture systems (73). In the cultivation of tilapia and grey mullet, *L. garvieae* infections are associated with morbidity rates of 70% to 100% (74, 75). In our study, although *L. garvieae* was the dominant bacterial taxon in both families, consistent with previous reports, its relative abundance in the susceptible family’s gut tissue (mean RA: 70%) was significantly higher than in the resistant family (mean RA: 20.18%). The genus *Enterococcus* comprises Gram-positive, facultative anaerobic bacteria commonly found in the digestive systems of humans, insects, and other animals (76). These bacteria are widely distributed and contribute to the gut microbiota of various hosts. Certain *Enterococcus* species are currently employed as probiotic strains alongside *Lactobacillus* and *Bifidobacterium* species (77). Both experimental and theoretical research have demonstrated that *Enterococcus* strains can be beneficial in managing conditions such as diarrhea, irritable bowel syndrome, and immune modulation when used as probiotics (78). In our study, the relative abundance of *Enterococcus casseliflavus* in the resistant lineage (mean RA: 0.51%) is significantly higher than that in the susceptible lineage (mean RA: 0.03%). This strain has been reported to exhibit probiotic potential and is capable of inducing the expression of IFN-λ genes and interferon-stimulated genes (ISGs) associated with antiviral functions (79). In conclusion, we hypothesize that the reduction in *L. garvieae*’s relative abundance, along with the concomitant increase in *Enterococcus casseliflavus*, may contribute to resistance against DIV1. It is important to note that while our study observed distinct differences in gut microbial composition between the resistant and susceptible families—specifically, a higher abundance of pathogenic bacteria in the susceptible group and potential probiotics in the resistant group—we did not perform experiments to disentangle the independent contribution of microbiota from viral effects on host mortality. Therefore, we cannot exclude the possibility that differences in microbial composition may have influenced the observed mortality rates, independently or synergistically with DIV1 infection. Future studies incorporating microbiota transplantation or gnotobiotic models could help clarify the causal role of gut microbiota in disease resistance.

The correlation analysis indicates that 19 microbial species show a negative correlation with most upregulated genes in the resistant family and a positive correlation with downregulated genes, suggesting that these microbes may play a key role in the resistance mechanism by regulating immune responses or metabolic pathways. In contrast, four microbial species (e.g., *Enterococcus casseliflavus*) show an opposite pattern, possibly promoting resistance by enhancing immune responses or metabolic processes. These microbes may interact with host-specific genes to regulate the host’s immune system or metabolic pathways, thereby improving the host’s resistance to DIV1. Furthermore, this study found that both microbial and host functional gene markers can effectively distinguish between resistant and susceptible families. Microbial markers provide a theoretical foundation for microbial intervention, indicating that adjusting the composition of the gut microbiota may help improve host immune function and reduce the risk of DIV1 infection. Host genes, such as CUB-serine protease and other functional genes, demonstrated excellent predictive ability in distinguishing resistant families, with an AUC value of 100%. These genes are likely critical to the host’s immune response to DIV1 and could serve as selection markers in aquaculture breeding to enhance resistance. Although the results demonstrate significant potential for application, several key issues require further investigation. Firstly, the specific functions of microbes and host genes in DIV1 resistance must be explored in depth, and their mechanisms experimentally validated. Future research should focus on understanding how microbes regulate host gene expression to enhance immune responses or metabolic processes. Additionally, using germ-free models or microbial transplantation techniques will aid in further elucidating the specific roles of these microbes in resistance. Longitudinal studies will help assess the stability and performance of these microbial and host gene markers under varying environmental conditions. This will provide more reliable evidence for their practical application in aquaculture breeding, thereby promoting resistance enhancement.

In summary, we characterized one resistant and one susceptible family of *M. rosenbergii* based on significant differences in their survival following the DIV1 challenge. This difference was further corroborated by disparities in muscle viral load, histopathological changes, and the number of viral genes identified in the host transcriptome. Functional annotation and enrichment analyses revealed that immune responses and energy metabolism are key contributors to DIV1 resistance. Under uninfected conditions, the gene expression profile of the resistant family indicated a stronger immune response and better metabolic adaptation. The elevated expression of these genes suggests that the resistant family may be pre-adapted to respond to potential infections. Additionally, we observed a higher abundance of the potential probiotic *Enterococcus casseliflavus* in the resistant family, while *L. garvieae* was more abundant in the susceptible family. Based on the findings from this study, we propose that further investigation into these genes, strains, and their regulatory networks will enhance our understanding of the underlying resistance mechanisms and yield valuable markers for resistance breeding in *M. rosenbergii*.

## Data Availability

The datasets presented in this study have been deposited in the Sequence Read Archive (SRA) at the National Center for Biotechnology Information (NCBI). The transcriptome data can be accessed using the accession number PRJNA1256795 (https://www.ncbi.nlm.nih.gov/bioproject/PRJNA1256795), while the microbiome sequencing data are available under the accession number PRJNA1256775 (https://www.ncbi.nlm.nih.gov/bioproject/PRJNA1256775).
