# Supplementary material for: Integrated transcriptomic and microbiomic analyses reveal mechanisms of Decapod iridescent virus 1 resistance in Macrobrachium rosenbergii
Source: Front Immunol. 2025 May 26;16:1611481. doi: 10.3389/fimmu.2025.1611481 (PMC12146183; doi:10.3389/fimmu.2025.1611481)
Supplement: Supplementary file 3 [file Table1.docx]

Supplementary Figure 1. Reads distribution in chromosomes.


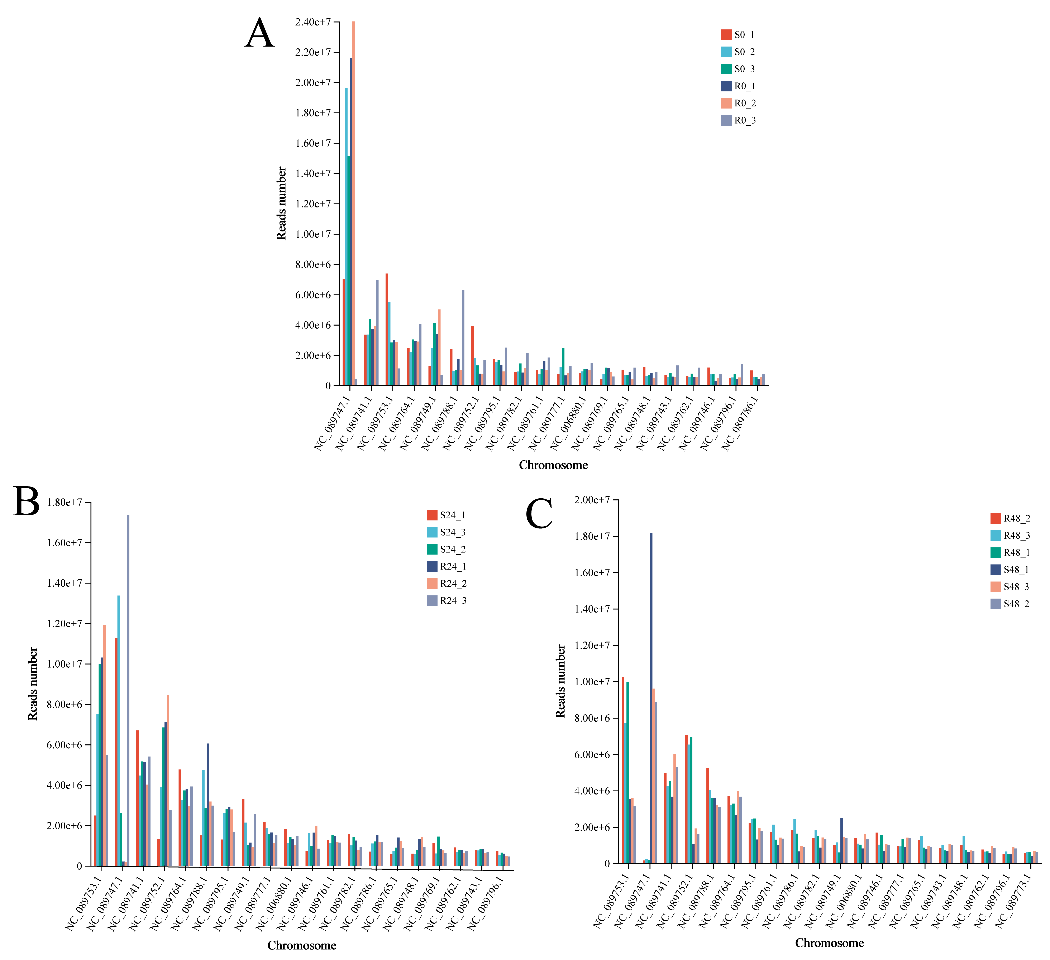


Supplementary Figure 2. Comparison of microbial diversity using the chao and simpson indices between susceptible (S2-2) and resistant (R27-1) families at 0 hpi (A and D), 24 hpi (B and E), and 48 hpi (C and F).


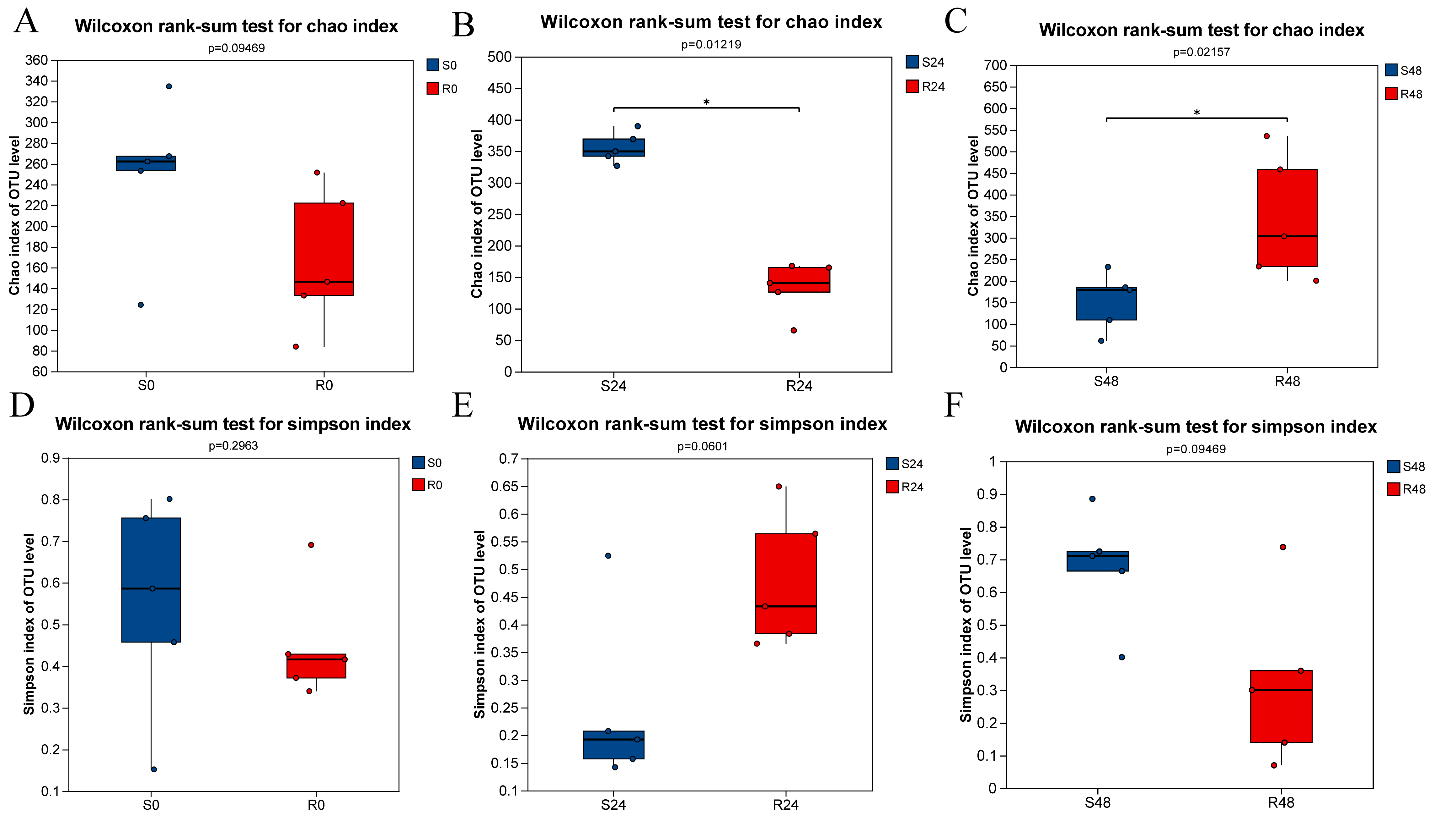


Table S1. qRT-PCR detection primers and probes.

| Primer Name | Primer and Probe Sequence (5′–3′) | Reference |
| --- | --- | --- |
| DIV1-qF | AGGAGAGGGAAATAACGGGAAAAC | [1] |
| DIV1-qR | CGTCAGCATTTGGTTCATCCATG |  |
| Probe | FAM-CTGCCCATCTAACACCATCTCCCGCCC-TAMRA |  |
| IPV-qF | AGGAGAGGGTTTTGGCTTG | [2] |
| IPV-qR | CTGGATTGGAAGGGAACTCTG |  |
| Probe | FAM-GAAGATGTCATCGTCCCAGAGTT-TAMRA |  |
| WSSV-qF | TGAGGTTGGATCAGGCTACTTC | [3] |
| WSSV-qR | CCGCATCTTCTTCCTTCATCTG |  |
| Probe | FAM-CAAGTACCCAGGCCCAGTGTCATACGTT-TAMRA |  |
| IHHNV-qF | TACTCCGGACACCCAACCA | [4] |
| IHHNV-qR | GGCTCTGGCAGCAAAGGTAA |  |
| Probe | FAM-ACCAGACATAGAGCTACAATCCTCGCCTATTTG-TAMRA |  |
| AHPND-qF | TIGGACTGTCGAACCAAACG | [5] |
| AHPND-qR | GCACCCCATIGGTATIGAATG |  |
| Probe | FAM-AGACAGCAAACATACACCTATCATCCCGGA-TAMRA |  |
| EHP-qF | AGTAAACTATGCCGACAA | [6] |
| EHP-qR | AATTAAGCAGCACAATCC |  |
| Probe | FAM-TCCTGGTAGTGTCCTTCCGT-TAMRA |  |
| DIV1_00007-qF | ATCGCTTTCACATTCCTCA | This study |
| DIV1_00007-qR | GGAAATGCAACAGCAGGTT |  |
| DIV1_00014-qF | GCAGCCGTCACAACACCA | This study |
| DIV1_00014-qR | GGCATTTGCGGGAATCGT |  |
| DIV1_00027-qF | CCAAACAGGGTGGTAAGAAC | This study |
| DIV1_00027-qR | AATCGTCCAGGTGTAGCG |  |
| DIV1_00038-qF | ACCGATTCATATTGGTTTGG | This study |
| DIV1_00038-qR | TTCTTTGCGGGTTGCTTT |  |
| DIV1_00053-qF | TCAAGGAAACCCAAATCG | This study |
| DIV1_00053-qR | TGCTATCGTAACCCAACCA |  |
| DIV1_00075-qF | TCTAAGAACCAAATGAACAGT | This study |
| DIV1_00075-qR | ATTCCTCCCGATCAACAA |  |
| DIV1_00084-qF | GACGAATGGGTTGGTGAT | This study |
| DIV1_00084-qR | GATCTGGGATTGGATGTTG |  |
| DIV1_00095-qF | GTCTGAACCAGCACCGAGTC | This study |
| DIV1_00095-qR | GGTTTCACGCTTCATATTCG |  |
| DIV1_00155-qF | CGGCCATCCATTCCCTCT | This study |
| DIV1_00155-qR | TCGGCTTCCAATTCCTCTTG |  |
| DIV1_00175-qF | CTCTTATTCATTTCGGTTGG | This study |
| DIV1_00175-qR | TGTCTGATTTCATTGTGGC |  |

|  |
| --- |

Table S2. Relative expression and functional annotation of viral genes detected in transcriptomic data.

| Gene_id | S48_1 | S48_2 | S48_3 | R48_1 | R48_2 | R48_3 | ORF | Pridicted function |
| --- | --- | --- | --- | --- | --- | --- | --- | --- |
| DIV1_00002 | 7072.3 | 0.0 | 0.0 | 0 | 0 | 0 | 154R |  |
| DIV1_00006 | 0.0 | 2244.8 | 1710.9 | 0 | 0 | 0 | 150R |  |
| DIV1_00007 | 47413.4 | 16852.6 | 99629.3 | 0 | 0 | 0 | 148R |  |
| DIV1_00008 | 0.0 | 16970.8 | 0.0 | 0 | 0 | 0 | 147R |  |
| DIV1_00009 | 28909.0 | 21715.4 | 8093.5 | 0 | 0 | 0 | 146R |  |
| DIV1_00010 | 0.0 | 1120.0 | 0.0 | 0 | 0 | 0 | 145R |  |
| DIV1_00011 | 0.0 | 2115.7 | 1613.1 | 0 | 0 | 0 | 144L |  |
| DIV1_00012 | 0.0 | 3978.1 | 0.0 | 0 | 0 | 0 | 143L |  |
| DIV1_00013 | 0.0 | 0.0 | 7416.3 | 0 | 0 | 0 | 142R |  |
| DIV1_00014 | 53617.1 | 24335.0 | 31373.5 | 0 | 0 | 1000000 | 141L |  |
| DIV1_00015 | 15359.3 | 15053.2 | 18160.9 | 0 | 0 | 0 | 140L |  |
| DIV1_00017 | 0.0 | 1641.9 | 4990.6 | 0 | 0 | 0 | 138R |  |
| DIV1_00019 | 7123.8 | 13039.0 | 6640.3 | 0 | 0 | 0 | 136R |  |
| DIV1_00020 | 0.0 | 3010.6 | 0.0 | 0 | 0 | 0 | 135L |  |
| DIV1_00022 | 0.0 | 2949.8 | 4500.0 | 0 | 0 | 0 | 133L |  |
| DIV1_00025 | 0.0 | 25364.3 | 0.0 | 0 | 0 | 0 | 129R |  |
| DIV1_00026 | 0.0 | 0.0 | 28638.4 | 0 | 0 | 0 | 128R |  |
| DIV1_00027 | 43887.1 | 32047.3 | 70655.2 | 0 | 0 | 0 | 127L | Ca2+-binding RTX toxin-related protein |
| DIV1_00032 | 12977.7 | 6537.4 | 19909.0 | 0 | 0 | 0 | 120L | hypothetical protein 1DG000109 |
| DIV1_00033 | 0.0 | 37014.9 | 0.0 | 0 | 0 | 0 | 119R |  |
| DIV1_00034 | 0.0 | 0.0 | 5228.9 | 0 | 0 | 0 | 118R |  |
| DIV1_00036 | 0.0 | 0.0 | 1074.1 | 0 | 0 | 0 | 116L |  |
| DIV1_00037 | 6301.7 | 0.0 | 0.0 | 0 | 0 | 0 | 115L | hypothetical protein 4TH000151 |
| DIV1_00038 | 34492.1 | 22806.5 | 20721.6 | 0 | 0 | 0 | 114L |  |
| DIV1_00039 | 0.0 | 13484.2 | 19557.4 | 0 | 0 | 0 | 113L |  |
| DIV1_00040 | 0.0 | 6354.8 | 0.0 | 0 | 0 | 0 | 112L | hypothetical protein KM509_gp042 |
| DIV1_00042 | 0.0 | 3060.1 | 2326.9 | 0 | 0 | 0 | 110R |  |
| DIV1_00043 | 0.0 | 17563.3 | 0.0 | 0 | 0 | 0 | 109R |  |
| DIV1_00044 | 0.0 | 6703.4 | 17825.5 | 0 | 0 | 0 | 108L |  |
| DIV1_00045 | 0.0 | 17744.9 | 3360.1 | 0 | 0 | 0 | 107L | ribonuclease III |
| DIV1_00048 | 0.0 | 5898.9 | 8780.7 | 0 | 0 | 0 |  | head decoration |
| DIV1_00049 | 18546.7 | 3600.2 | 10822.6 | 0 | 0 | 0 | 104R | hypothetical protein KM509_gp050 |
| DIV1_00051 | 0.0 | 2008.8 | 3046.6 | 0 | 0 | 0 | 102L |  |

Table S2 (continued)

| Gene_id | S48_1 | S48_2 | S48_3 | R48_1 | R48_2 | R48_3 | ORF | Pridicted function |
| --- | --- | --- | --- | --- | --- | --- | --- | --- |
| DIV1_00053 | 0.0 | 0.0 | 45542.8 | 0 | 0 | 0 | 100L |  |
| DIV1_00055 | 3179.0 | 2927.7 | 2988.8 | 0 | 0 | 0 | 097L | DNA-dependent RNA polymerase II largest subunit |
| DIV1_00058 | 23128.1 | 0.0 | 6624.5 | 0 | 0 | 0 | 094R | hypothetical protein KM509_gp059 |
| DIV1_00059 | 0.0 | 0.0 | 2098.6 | 0 | 0 | 0 | 093L |  |
| DIV1_00061 | 0.0 | 32471.7 | 0.0 | 0 | 0 | 0 | 091R |  |
| DIV1_00064 | 0.0 | 900.1 | 0.0 | 0 | 0 | 0 | 088L | NTPase helicase |
| DIV1_00065 | 0.0 | 0.0 | 2365.3 | 0 | 0 | 0 | 087L | NAD-dependent DNA ligase |
| DIV1_00066 | 0.0 | 27133.7 | 23268.7 | 0 | 0 | 0 | 086L |  |
| DIV1_00069 | 0.0 | 1447.9 | 4405.9 | 0 | 0 | 0 | 084L | hypothetical protein 4TH000124 |
| DIV1_00070 | 0.0 | 776.1 | 1187.9 | 0 | 0 | 0 | 083L | DNA-directed RNA polymerase II second largest subunit |
| DIV1_00072 | 0.0 | 0.0 | 9645.1 | 0 | 0 | 0 | 081R |  |
| DIV1_00075 | 418886.8 | 0.0 | 0.0 | 0 | 0 | 0 | 078R |  |
| DIV1_00076 | 30243 | 0.0 | 0.0 | 0 | 0 | 0 | 077L |  |
| DIV1_00077 | 0.0 | 15464.5 | 15384.3 | 0 | 0 | 0 | 076L |  |
| DIV1_00079 | 0.0 | 3417.7 | 0.0 | 0 | 0 | 0 | 074L | hypothetical protein KM509_gp078 |
| DIV1_00080 | 0.0 | 4287.4 | 8171.6 | 0 | 0 | 0 | 073R | hypothetical protein 4TH000114 |
| DIV1_00084 | 65525.3 | 242940 | 198801 | 0 | 0 | 0 | 068R |  |
| DIV1_00086 | 0.0 | 2966.5 | 0.0 | 0 | 0 | 0 | 066R |  |
| DIV1_00088 | 0.0 | 584.2 | 893.2 | 0 | 0 | 0 | 064L |  |
| DIV1_00089 | 0.0 | 9589.5 | 0.0 | 0 | 0 | 0 | 063L |  |
| DIV1_00094 | 0.0 | 0.0 | 671.8 | 0 | 0 | 0 | 061L |  |
| DIV1_00095 | 65525.3 | 0.0 | 9036.4 | 0 | 0 | 0 | 060L |  |
| DIV1_00097 | 0.0 | 4682.2 | 0.0 | 0 | 0 | 0 | 058L |  |
| DIV1_00098 | 4703.7 | 3812.6 | 4362.9 | 0 | 0 | 0 | 057R |  |
| DIV1_00099 | 0.0 | 1044.2 | 0.0 | 0 | 0 | 0 | 056R |  |
| DIV1_00101 | 0.0 | 19987.7 | 9896.1 | 0 | 0 | 0 | 054L |  |
| DIV1_00102 | 0.0 | 19381.2 | 5817.2 | 0 | 0 | 0 | 053L |  |

Table S2 (continued)

| Gene_id | S48_1 | S48_2 | S48_3 | R48_1 | R48_2 | R48_3 | ORF | Pridicted function |
| --- | --- | --- | --- | --- | --- | --- | --- | --- |
| DIV1_00103 | 23617.1 | 9535.1 | 23609.2 | 0 | 0 | 0 | 052L | hypothetical protein KM509_gp099 |
| DIV1_00105 | 0.0 | 1095.1 | 1669.5 | 0 | 0 | 0 | 050L | hypothetical protein KM509_gp102 |
| DIV1_00106 | 0.0 | 3939.3 | 2988.0 | 0 | 0 | 0 | 049R |  |
| DIV1_00108 | 0.0 | 0.0 | 5835.4 | 0 | 0 | 0 | 047L |  |
| DIV1_00110 | 32229.3 | 0.0 | 4748.7 | 0 | 0 | 0 | 045R |  |
| DIV1_00112 | 0.0 | 10441.4 | 0.0 | 0 | 0 | 0 | 043R |  |
| DIV1_00113 | 0.0 | 838.0 | 0.0 | 0 | 0 | 0 | 042L |  |
| DIV1_00115 | 0.0 | 8035.2 | 0.0 | 0 | 0 | 0 | 040L |  |
| DIV1_00117 | 0.0 | 8061.5 | 0.0 | 0 | 0 | 0 | 038R |  |
| DIV1_00118 | 0.0 | 971.9 | 743.4 | 0 | 0 | 0 | 037L | DNA primase |
| DIV1_00120 | 0.0 | 0.0 | 5817.2 | 0 | 0 | 0 | 035R |  |
| DIV1_00122 | 0.0 | 1904.7 | 2890.5 | 0 | 0 | 0 | 033L | hypothetical protein KM509_gp118, partial |
| DIV1_00123 | 0.0 | 7040.5 | 0.0 | 0 | 0 | 0 | 032R |  |
| DIV1_00125 | 0.0 | 13484.2 | 0.0 | 0 | 0 | 0 | 029L |  |
| DIV1_00126 | 0.0 | 19630.5 | 0.0 | 0 | 0 | 0 | 028R |  |
| DIV1_00127 | 0.0 | 11446.1 | 8638.0 | 0 | 0 | 0 | 027R | hypothetical protein 1DG000025 |
| DIV1_00129 | 0.0 | 1095.1 | 0.0 | 0 | 0 | 0 | 025L |  |
| DIV1_00131 | 0.0 | 14341.7 | 0.0 | 0 | 0 | 0 | 024L |  |
| DIV1_00132 | 0.0 | 21410.7 | 26822.7 | 0 | 0 | 0 | 023L |  |
| DIV1_00134 | 0.0 | 2923.6 | 0.0 | 0 | 0 | 0 | 021L |  |
| DIV1_00135 | 0.0 | 7013.1 | 0.0 | 0 | 0 | 0 | 020L |  |
| DIV1_00140 | 0.0 | 7247.0 | 0.0 | 0 | 0 | 0 | 015R | N-acetylmuramoyl-L-alanine amidase |
| DIV1_00141 | 8896.3 | 3558.1 | 5403.5 | 0 | 0 | 0 | 014L |  |
| DIV1_00142 | 0.0 | 7792.5 | 0.0 | 0 | 0 | 0 | 013L |  |
| DIV1_00143 | 0.0 | 0.0 | 6998.1 | 0 | 0 | 0 | 012L |  |
| DIV1_00147 | 0.0 | 0.0 | 10219.5 | 0 | 0 | 0 | 007L |  |
| DIV1_00149 | 0.0 | 0.0 | 18850.6 | 0 | 0 | 0 | 005R |  |
| DIV1_00151 | 0.0 | 7912.8 | 3986.1 | 0 | 0 | 0 | 003L |  |

Table S2 (continued)

| Gene_id | S48_1 | S48_2 | S48_3 | R48_1 | R48_2 | R48_3 | ORF | Pridicted function |
| --- | --- | --- | --- | --- | --- | --- | --- | --- |
| DIV1_00153 | 0.0 | 2149.4 | 3277.3 | 0 | 0 | 0 | 001R, partial | major capsid protein, partial |
| DIV1_00155 | 9144.6 | 21927.5 | 22193.7 | 0 | 0 | 0 | 177L |  |
| DIV1_00156 | 0.0 | 7330.9 | 16522.1 | 0 | 0 | 0 | 176L |  |
| DIV1_00157 | 12365.5 | 3741.0 | 0.0 | 0 | 0 | 0 | 175R | NTPase helicase |
| DIV1_00160 | 0.0 | 0.0 | 4977.3 | 0 | 0 | 0 | 172R |  |
| DIV1_00164 | 0.0 | 1888.5 | 4322.4 | 0 | 0 | 0 | 168L |  |
| DIV1_00167 | 0.0 | 4155.0 | 18677.2 | 0 | 0 | 0 | 165L | hypothetical protein 4TH000035 |
| DIV1_00168 | 0.0 | 863.6 | 0.0 | 0 | 0 | 0 | 164L |  |
| DIV1_00170 | 0.0 | 8396.2 | 0.0 | 0 | 0 | 0 | 162R |  |
| DIV1_00171 | 0.0 | 5985.0 | 8906.7 | 0 | 0 | 0 | 161R | hypothetical protein 1DG000145 |
| DIV1_00172 | 0.0 | 0.0 | 11330.2 | 0 | 0 | 0 | 160L |  |
| DIV1_00173 | 9685.1 | 0.0 | 2931.5 | 0 | 0 | 0 | 159L |  |
| DIV1_00174 | 0.0 | 0.0 | 6907.2 | 0 | 0 | 0 | 158R | hypothetical protein KM509_gp168 |
| DIV1_00175 | 17171.1 | 13748.7 | 23497.1 | 0 | 0 | 0 | 157L | myristylated membrane |
| DIV1_00176 | 0.0 | 41065.9 | 0.0 | 0 | 0 | 0 | 156L | hypothetical protein KM509_gp170 |

Table S3. Sequencing data statistics.

| Sample | Raw reads | Raw bases | Clean reads | Clean bases | Error rate (%) | Q20 (%) | Q30 (%) | GC content (%) |
| --- | --- | --- | --- | --- | --- | --- | --- | --- |
| R0_1 | 44123516 | 6.66E+09 | 43740078 | 6.55E+09 | 0.0118 | 98.86 | 96.32 | 44.45 |
| R0_2 | 48623384 | 7.34E+09 | 48205588 | 7.22E+09 | 0.0118 | 98.89 | 96.43 | 43.49 |
| R0_3 | 49264996 | 7.44E+09 | 48826004 | 7.3E+09 | 0.0119 | 98.79 | 96.21 | 43.42 |
| S0_1 | **42051634** | 6.35E+09 | **41699334** | 6.24E+09 | 0.0118 | 98.85 | 96.32 | 44.3 |
| S0_2 | 46194014 | 6.98E+09 | 45783276 | 6.86E+09 | 0.0118 | 98.86 | 96.37 | 43.44 |
| S0_3 | 51200432 | 7.73E+09 | 50748188 | 7.61E+09 | 0.0118 | 98.83 | 96.3 | 43.26 |
| R24_1 | 51865676 | 7.83E+09 | 51484730 | 7.72E+09 | 0.0118 | 98.86 | 96.36 | 44.51 |
| R24_2 | 47373014 | 7.15E+09 | 47038054 | 7.05E+09 | 0.0118 | 98.89 | 96.42 | 44.69 |
| R24_3 | 49794782 | 7.52E+09 | 49375982 | 7.4E+09 | 0.0118 | 98.87 | 96.39 | 44.03 |
| S24_1 | 48602500 | 7.34E+09 | 48241222 | 7.22E+09 | 0.0118 | 98.84 | 96.32 | 43.01 |
| S24_2 | 51216838 | 7.73E+09 | 50864536 | 7.62E+09 | 0.0118 | 98.88 | 96.41 | 44.08 |
| S24_3 | 51456814 | 7.77E+09 | 51123424 | 7.67E+09 | 0.0117 | 98.91 | 96.48 | 44.13 |
| R48_1 | 44724010 | 6.75E+09 | 44298666 | 6.64E+09 | 0.0119 | 98.83 | 96.27 | 44.32 |
| R48_2 | 47643152 | 7.19E+09 | 47196160 | 7.07E+09 | 0.0119 | 98.83 | 96.28 | 44.81 |
| R48_3 | **52033436** | 7.86E+09 | **51644420** | 7.75E+09 | 0.0118 | 98.85 | 96.32 | 44.88 |
| S48_1 | 43492804 | 6.57E+09 | 43116192 | 6.47E+09 | 0.0118 | 98.85 | 96.31 | 44 |
| S48_2 | 46436476 | 7.01E+09 | 46028854 | 6.9E+09 | 0.0119 | 98.81 | 96.23 | 43.64 |
| S48_3 | 50614310 | 7.64E+09 | 50185614 | 7.52E+09 | 0.0119 | 98.8 | 96.22 | 43.45 |

Note:(1) Sample: Sample name; (2) Raw reads: The total number of raw sequencing entries; (3) Raw bases: The total amount of raw sequencing data (i.e., the number of raw reads multiplied by the read length); (4) Clean reads: The total number of reads after quality control; (5) Clean bases: The total amount of sequencing data after quality control (i.e., the number of clean reads multiplied by the read length); (6) Error rate (%): The average error rate of sequencing bases in the quality-controlled data, generally below 0.1%; (7) Q20 (%), Q30 (%): Quality assessment of the quality-controlled sequencing data, where Q20 and Q30 refer to the percentage of bases with sequencing quality above 99% and 99.9%, respectively; (8) GC content (%): The percentage of G and C bases in the quality-controlled data relative to the total number of bases.

Table S4. Alignment results statistics.

| Sample | Total reads | Total mapped | Multiple mapped | Uniquely mapped |
| --- | --- | --- | --- | --- |
| R0_1 | 43740078 | 41532547 (94.95%) | 8040609 (18.38%) | 33491938 (76.57%) |
| R0_2 | 48205588 | 45915596 (95.25%) | 7731055 (16.04%) | 38184541 (79.21%) |
| R0_3 | 48826004 | 45337711 (92.86%) | 6372744 (13.05%) | 38964967 (79.8%) |
| S0_1 | 41699334 | 39320715 (94.3%) | 5864111 (14.06%) | 33456604 (80.23%) |
| S0_2 | 45783276 | 43484354 (94.98%) | 7329936 (16.01%) | 36154418 (78.97%) |
| S0_3 | 50748188 | 47871174 (94.33%) | 6971119 (13.74%) | 40900055 (80.59%) |
| R24_1 | 51484730 | 48675173 (94.54%) | 8252351 (16.03%) | 40422822 (78.51%) |
| R24_2 | 47038054 | 44330609 (94.24%) | 6985583 (14.85%) | 37345026 (79.39%) |
| R24_3 | 49375982 | 47094830 (95.38%) | 9093509 (18.42%) | 38001321 (76.96%) |
| S24_1 | 48241222 | 45787720 (94.91%) | 8097508 (16.79%) | 37690212 (78.13%) |
| S24_2 | 50864536 | 48052378 (94.47%) | 7839428 (15.41%) | 40212950 (79.06%) |
| S24_3 | 51123424 | 48541271 (94.95%) | 8629161 (16.88%) | 39912110 (78.07%) |
| R48_1 | 44298666 | 42011633 (94.84%) | 6910005 (15.6%) | 35101628 (79.24%) |
| R48_2 | 47196160 | 44584560 (94.47%) | 7814773 (16.56%) | 36769787 (77.91%) |
| R48_3 | 51644420 | 48301899 (93.53%) | 7141933 (13.83%) | 41159966 (79.7%) |
| S48_1 | 43116192 | 40948366 (94.97%) | 7370131 (17.09%) | 33578235 (77.88%) |
| S48_2 | 46028854 | 43300156 (94.07%) | 6822168 (14.82%) | 36477988 (79.25%) |
| S48_3 | 50185614 | 47240484 (94.13%) | 7486337 (14.92%) | 39754147 (79.21%) |

Note: (1) Sample: Sample name; (2) Total reads: The number of sequences after filtering the sequencing data (i.e., Clean reads); (3) Total mapped: The number of Clean reads mapped to the genome; (4) Multiple mapped: The number of Clean reads mapped to multiple locations on the reference sequence; (5) Uniquely mapped: The number of Clean reads mapped to a unique location on the reference sequence.

Table S5. Reads distribution statistics.

| Sample | CDS | Intergenic | Introns | 3’UTR | 5’UTR |
| --- | --- | --- | --- | --- | --- |
| R0_1 | 34956739.0 (58.72%) | 856415.0 (1.44%) | 2154926.0 (3.62%) | 16013562.0 (26.9%) | 5551954.0 (9.33%) |
| R0_2 | 37179276.0 (58.76%) | 1035736.0 (1.64%) | 2474402.0 (3.91%) | 17336123.0 (27.4%) | 5244616.0 (8.29%) |
| R0_3 | 40202201.0 (62.23%) | 2138257.0 (3.31%) | 3695337.0 (5.72%) | 11855306.0 (18.35%) | 6713131.0 (10.39%) |
| S0_1 | 35876272.0 (64.97%) | 923886.0 (1.67%) | 2124055.0 (3.85%) | 11019037.0 (19.96%) | 5275457.0 (9.55%) |
| S0_2 | 33961151.0 (56.93%) | 1101796.0 (1.85%) | 2096255.0 (3.51%) | 16760707.0 (28.1%) | 5733018.0 (9.61%) |
| S0_3 | 38763907.0 (58.62%) | 1470826.0 (2.22%) | 3142375.0 (4.75%) | 16294085.0 (24.64%) | 6456819.0 (9.76%) |
| R24_1 | 46953302.0 (68.11%) | 1170569.0 (1.7%) | 3066188.0 (4.45%) | 10755124.0 (15.6%) | 6988239.0 (10.14%) |
| R24_2 | 44135655.0 (70.89%) | 839254.0 (1.35%) | 2432469.0 (3.91%) | 8866908.0 (14.24%) | 5982861.0 (9.61%) |
| R24_3 | 40405407.0 (61.87%) | 1012410.0 (1.55%) | 2907412.0 (4.45%) | 14197004.0 (21.74%) | 6781619.0 (10.38%) |
| S24_1 | 36384622.0 (58.44%) | 1416233.0 (2.27%) | 3516705.0 (5.65%) | 13953753.0 (22.41%) | 6985278.0 (11.22%) |
| S24_2 | 44306781.0 (66.3%) | 1281982.0 (1.92%) | 2950731.0 (4.42%) | 11431368.0 (17.11%) | 6856766.0 (10.26%) |
| S24_3 | 42524242.0 (63.1%) | 1051294.0 (1.56%) | 2668111.0 (3.96%) | 14311278.0 (21.24%) | 6836352.0 (10.14%) |
| R48_1 | 39388183.0 (67.39%) | 921939.0 (1.58%) | 2636550.0 (4.51%) | 9335600.0 (15.97%) | 6167477.0 (10.55%) |
| R48_2 | 43311209.0 (68.87%) | 990175.0 (1.57%) | 2790705.0 (4.44%) | 9508456.0 (15.12%) | 6291117.0 (10.0%) |
| R48_3 | 46845429.0 (67.27%) | 1348274.0 (1.94%) | 2724508.0 (3.91%) | 12512828.0 (17.97%) | 6210617.0 (8.92%) |
| S48_1 | 34316197.0 (59.45%) | 892515.0 (1.55%) | 2175753.0 (3.77%) | 14664114.0 (25.4%) | 5675753.0 (9.83%) |
| S48_2 | 37256562.0 (61.58%) | 1343266.0 (2.22%) | 3007842.0 (4.97%) | 12562122.0 (20.76%) | 6330568.0 (10.46%) |
| S48_3 | 40055289.0 (61.25%) | 1487109.0 (2.27%) | 3280963.0 (5.02%) | 13826208.0 (21.14%) | 6746724.0 (10.32%) |

Note: The distribution of reads across different regions of the reference genome. CDS: Coding sequence; Intergenic: Intergenic region; Introns: Intron region; 3’UTR, 5’UTR: 3’ or 5’ untranslated region.

Reference

[1] Qiu L, Chen MM, Wan XY, Zhang QL, Li C, Dong X, et al. Detection and quantification of shrimp hemocyte iridescent virus by TaqMan probe based real-time PCR. J Invertebr Pathol 2018;154:95–101. https://doi.org/10.1016/j.jip.2018.04.005.

[2] Wang G-H, Guo X-M, Huang X, Wang D-H, Chen Y-J, Qin J-H, et al. The quantitative characteristics of infection with infectious precocity virus (IPV) revealed with a new TaqMan probe-based real-time RT-PCR method. Aquaculture 2023;566:739179.

[3] Yang L-W, Wang Z-A, Geng R, Deng H-W, Niu S, Zuo H-L, et al. White spot syndrome virus (WSSV) inhibits hippo signaling and activates Yki to promote its infection in *Penaeus vannamei*. Microbiol Spectr 2022;11:e02363. https://doi.org/10.1128/spectrum.02363-22.

[4] Tang KF, Lightner DV. Detection and quantification of infectious hypodermal and hematopoietic necrosis virus in penaeid shrimp by real-time PCR. Dis Aquat Organ 2001;44:79–85. https://doi.org/10.3354/dao044079.

[5] Wan XY, Yang B, Huang J, Zhang QL, Xie GX, Wang HL, et al. Code of diagnosis for acute hepatopancreatic necrosis disease 2020.

[6] Liu Y-M, Qiu L, Sheng A-Z, Wan X-Y, Cheng D-Y, Huang J. Quantitative detection method of *Enterocytozoon hepatopenaei* using TaqMan probe real-time PCR. J Invertebr Pathol 2018;151:191–6. https://doi.org/10.1016/j.jip.2017.12.006.
